# Composite Fish Collagen-Hyaluronate Based Lyophilized Scaffolds Modified with Sodium Alginate for Potential Treatment of Chronic Wounds

**DOI:** 10.3390/polym14081550

**Published:** 2022-04-11

**Authors:** Meena Afzali, Joshua Siaw Boateng

**Affiliations:** School of Science, Faculty of Engineering and Science, University of Greenwich, Kent ME4 4TB, UK; m.afzali@greenwich.ac.uk

**Keywords:** chronic wounds, fish collagen, hyaluronic acid, scaffolds, sodium alginate

## Abstract

Chronic wounds are characterized by both decreased collagen deposition and increased collagen breakdown. It is reasonable to hypothesize that exogenous collagen can potentially promote wound healing by reducing degradation enzymes in the wound environment and disrupting the cycle of chronicity. Therefore, this study aimed to develop an optimal combination of fish collagen (FCOL), sodium alginate (SA), and hyaluronic acid (HA) loaded with bovine serum albumin (BSA) as a model protein fabricated as lyophilized scaffolds. The effects of sodium alginate (SA#) with higher mannuronic acid (M) were compared to sodium alginate (SA*) with higher guluronic acid (G). The SA* with higher G resulted in elegant scaffolds with hardness ranging from 3.74 N–4.29 N that were able to withstand the external force due to the glycosidic bonds in guluronic acid. Furthermore, the high G content also had a significant effect on the pore size, pore shape, and porosity. The water absorption (WA) ranged from 380–1382 (%) and equilibrium water content (EWC) 79–94 (%) after 24 h incubation at 37 °C. The SA* did not affect the water vapor transmission rate (WVTR) but incorporating BSA significantly increased the WVTR making these wound dressing scaffolds capable of absorbing about 50% exudate from a heavily exuding chronic wound. The protein released from the composite systems was best explained by the Korsmeyer–Peppas model with regression R^2^ values ranging from 0.896 to 0.971 and slope or *n <* 0.5 indicating that the BSA release mechanism was governed by quasi-Fickian diffusion. Cell viability assay showed that the scaffolds did not inhibit the proliferation of human dermal fibroblasts and human epidermal keratinocytes, and are therefore biocompatible. In vitro blood analysis using human whole blood confirmed that the BSA-loaded SA*:FCOL:HA scaffolds reduced the blood clotting index (BCI) by up to 20% compared to a commercially available sponge for chronic wounds. These features confirm that SA*:FCOL:HA scaffolds could be applied as a multifunctional wound dressing.

## 1. Introduction

Regeneration and tissue repair is a complex biological process comprising several overlapping and highly integrated molecular and cellular events. Each stage of this process involves the interplay between different specialized cells such as platelets, macrophages, fibroblasts, and keratinocytes. Further, wound healing is also influenced by the action of essential biomolecules such as glycoproteins, cytokines, chemokines, growth factors, proteases, and endocrine hormones [1]. When an injury occurs, a series of orchestrated events are initiated aiming to prevent infection and repair the wounded tissue. These phases include hemostasis, inflammation, fibroblast proliferation, cell contraction, angiogenesis, and re-epithelialization, which results in scar tissue formation and wound remodeling [2]. Chronic wounds fail to proceed through an orderly and timely self-healing process and typically take longer than 3 months to heal. Alteration in the activity of the immune cells to control bacterial infection and diminished vascularization are some of the underlying challenges that inhibit the physiologic healing of chronic wounds and thereby inhibit the repair and deposition of the extracellular matrix (ECM) [3].

Prolonged inflammation subsequently causes tissue destruction and life-threatening complications such as lower extremity amputations, which cause permanent disabilities and pain for patients [3]. Chronic wounds are an important and expensive medical challenge, which represent a significant burden on the patient and the National Health Service (NHS). Trends in the UK population over the next 20 years are expected to result in a significant increase in the number of patients with chronic wounds. There is therefore increased interest in cost effective, advanced dressing devices that can accelerate the healing of chronic wounds by targeting different aspects of the wound healing microenvironment to improve patients’ quality of life and reduce health care costs [4].

Collagen, secreted by fibroblasts, is the most prevalent protein in the ECM. It accounts for three-quarters of dry skin and plays a vital role in each phase of wound healing, and due to its chemotactic role, it attracts cells such as fibroblasts and keratinocytes to the wound. The dynamic creation and destruction of collagen components are normal events in acute wounds, however, in chronic wounds, the deposition of de novo collagen is delayed or prevented by many factors [5]. Environmental factors also contribute to decreased collagen in the chronic wound bed including the levels of matrix metalloproteinases (MMPs) and elastase. MMPs are involved in the proteolytic degradation of native intact collagen and partially degraded fragments of collagen are also elevated in chronic wounds [6,7]. Therefore, the presence of collagen in wound dressings is highly advantageous to promote wound healing by encouraging the migration of macrophages and fibroblasts to the wound site. Additionally, collagen dressings promote the development of new blood vessels and the migration of keratinocytes, thus contributing to wound re-epithelialization [8]. Dressings containing exogenous collagen will act as a substrate to attract elastase and MMPs thereby inhibiting the degradation of newly formed native collagen in the wounded area and can therefore potentially promote wound healing [9,10]. Type I collagen used in a biomedical application has been successfully extracted from bovine and porcine sources, however, animal-derived collagen is associated with religious constraints, potential allergies, and concerns of transmissible diseases.

Clinical studies in rodent models have shown that FCOL promotes wound healing. Due to high biodegradability, biocompatibility, and low immunogenicity, FCOL is considered favorable for biomedical applications including wound healing [11,12]. While being susceptible to both acute and chronic wounds, fish have quicker healing capability compared to humans. Although fish skin and human skin are structurally similar the difference in the rate of healing is believed to be due to the amino acid composition in collagen, which plays an important role in the enzymatic process of wound healing. The quantity and composition of amino acids and collagen differ between fish and mammalians, for example, FCOL has a lower hydroxyproline content that causes lower denaturation temperature, thus making it easier to be absorbed due to its low molecular weight. Some studies have also highlighted that FCOL contains higher quantities of arginine and glycine that are known for their wound healing and anti-inflammatory properties [13,14].

Although FCOL is reported to have the capability to heal wounds faster, differences in its amino acid composition compared to bovine collagen and the harsh extraction and manufacturing processes affect its thermal, structural, and mechanical properties, and covalent crosslinks. This suggests the need for enhancing cross-links within its matrix by combining them with other known biomaterials prior to use in wound dressings [15].

Therefore, this study aimed to develop novel biomaterial-based scaffolds with an optimal combination of FCOL, hyaluronic acid (HA); (a large glycosaminoglycan, an essential component of the ECM), and sodium alginate (SA) loaded with bovine serum albumin (BSA) as a model protein. To enhance the mechanical stability of FCOL:HA gels, and ultimately reduce the frequency of dressing changes, the effects of SA# with higher mannuronic acid (M) were also compared to SA* with higher guluronic acid (G) [10,11]. The G-block is stiffer and more extended in chain configuration than the M-block due to a higher degree of hindered rotation around the glycosidic linkages. Hence the mechanical properties of alginate gel are enhanced by the higher composition of G/M blocks and the length of the G blocks [12]. It is anticipated that reducing dressing change frequency will reduce the associated trauma and contribute to improving patient compliance.

## 2. Materials and Methods

### 2.1. Materials

Fish collagen type I (FCOL) was purchased from Creative Enzymes Inc. (Shirley, NY, USA), hyaluronic acid (HA) was purchased from Wisapple Biotech Co, Ltd. (Beijing, China), Pronatal^®^ LF 10/60 SA (SA 10/60) with G/M % ratios of 70/30 was kindly gifted by IMCD UK Limited, (Surrey, UK), sodium alginate (SA) with G/M % ratio 39/61, Gelatin (GEL) and D-mannitol (D-mann) were purchased from Sigma-Aldrich (Gillingham, UK)., bovine serum albumin (BSA), and calcium chloride were obtained from Acros Organics (Branchburg, NJ, USA) while sodium chloride (NaCl) was purchased from Fisher Scientific, (Loughborough, UK). Adult human primary epidermal keratinocytes [PCS-200-011, ATCC], human dermal fibroblasts [PCS-200-011, ATCC], dermal cell basal medium [PCS-200-030, ATCC], keratinocytes growth kit [PCS-200-040, ATCC], ATCC and Dulbecco’s Modified Eagle’s Medium (DMEM) [PCS-200-030, ATCC] were purchased from LGC standards (Middlesex, UK). Methyl thiazolyldiphenyl-tetrazolium bromide (MTT) and trypan blue stain were obtained from Thermo Fisher Scientific (Paisley, UK), fetal bovine serum was purchased from Sigma Aldrich, (Dorset, UK).

### 2.2. Methods

Preliminary formulation development studies were performed by varying the ratios of the polymers to identify the optimum concentrations of polymers required to produce stable aqueous gels of FCOL:HA appropriate for further formulation development. The composite polymeric gels of FCOL and HA were prepared by mixing and stirring the different ratios of polymers (Table 1) in 100 mL of distilled water (room temperature) on a magnetic stirrer at 750 rpm.

Once the polymers were adequately hydrated, the stirring speed was decreased to 500 rpm, and the mixture was left to stir for 2 h to allow complete dispersion during gel preparation to form a uniform gel. To overcome the weak mechanical properties of the initial ‘2-polymer’ (FCOL:HA) formulations caused by the brittle nature of FCOL, new composite gels comprising FCOL, HA, and SA were prepared. To obtain these ‘3-polymer’ (SA-FCOL-HA) composite gels, the concentration (ratio) of HA in the initial composite formulations was kept constant while that of FCOL was reduced, and the difference was made up with different grades of SA (as a modifier) as shown in (Table 1).

### 2.3. Preparation of Lyophilized Scaffolds

3 g of the prepared gel was poured into each mold of twelve-well plates (diameter 22.1 mm) (Thermo Fisher Scientific, Leicester, UK). The gels were freeze-dried in a Virtis Advantage XL 70 freeze dryer (Biopharma Process Systems, Winchester, UK), using an automated freeze-drying cycle as previously reported [16]. The samples were cooled from room temperature to 5 °C for 30 min, 5 °C to −5 °C for 1 h, and −5 °C to −50 °C for 3 h. Furthermore, an annealing step was incorporated in the freezing step to enhance the pore size distribution by increasing the temperature from −55 °C to −25 °C for 2.5 h and cooling it back to −55 °C for 3 h. In the primary drying stage, a pressure of 50 mTorr was applied and the temperature was increased from −55 °C to −25 °C for 12 h and further increased from −25 °C to +20 °C for 7 h. The same pressure was applied during secondary drying and the temperature was held at +20 °C for 6 h to remove the remaining residual moisture [16]. For the BSA-loaded scaffolds, the optimized gels were loaded with 75 µg/g of BSA and 20% D-mann (of total polymer weight). The lyophilized scaffolds were stored in a desiccator over silica gel to maintain a low moisture content that is ideal for maintaining protein stability until required for further analysis.

### 2.4. Physico-Chemical Characterization

#### 2.4.1. Scanning Electron Microscopy (SEM)

The surface morphology of the lyophilized scaffolds was analyzed using a (Hitachi SU8000, HI-0210-0005, Hitachi High-technologies; Düsseldorf, Germany) scanning electron microscope at an accelerating voltage of 1 kV and working distances of 8500 and 11,400 mm. A thin piece of the scaffold was fixed onto an aluminum stub with the aid of double-sided carbon tape and gold-coated, before the acquisition of the micrographs at magnifications of ×70 and ×9 k. The pore size and wall thickness of both the top and bottom of the scaffolds were measured for further analysis.

#### 2.4.2. X-ray Diffraction (XRD)

The physical form of the scaffolds and the starting materials was analyzed using a D8 Advance X-ray diffractometer (Bruker AXS GmbH, Karlsure, Germany) equipped with an exit slit of 0.6 mm and sample rotation of 30 rpm in 2-theta geometry transmission mode. The samples were compressed using a pair of clean compression glasses to 0.5 mm and mounted onto the sample holder using Mylar. The transmission diffractograms were acquired using the DIFFRAC plus XRD commander with a start to end angle from 2θ of 5° to 45°, step size of 0.02, the scan speed of 0.4 s at 40 kV and 40 mA with Cu Kα radiation.

#### 2.4.3. Attenuated Total Reflectance Fourier Transform Infrared Spectroscopy (ATR-FTIR)

To characterize the interactions between the polymers and between the polymers and BSA, FTIR spectrophotometer (Thermo Nicolet, Thermo Scientific, Basingstoke, UK) combined with ZnSe attenuated total reflectance (ATR) crystal accessory was used. A small quantity of the scaffold and powder (starting materials) was placed on the ATR crystal and pressed by the pressure clamp to allow proper contact between the sample and the ATR crystal. The spectra for the samples were collected at a resolution of 4 cm^−1^ and wavenumber range from 650–4000 cm^−1^.

#### 2.4.4. Texture Analysis (TA)

##### Mechanical Strength (Hardness)

Resistance to compressive deformation (hardness) and ease of recovery, representing the mechanical strength of the scaffolds was studied with a TA HD texture analyzer (Stable Microsystem Ltd., Surrey, UK) fitted with Texture Exponent 32 software program. The investigations were performed in compression mode using a 6 mm (P/6) cylindrical stainless-steel probe (Stable Microsystem Ltd.). To investigate the effect of total polymer content, the scaffolds (*n* = 3) were compressed at three different positions on both sides to a depth of 2 mm, using a trigger force of 0.001 N, at a speed of 1 mm/s and a 10 mm return distance.

##### Adhesion

The same texture analyzer mentioned above was used to study the adhesive performance of the scaffolds. The scaffolds (*n* = 3) were attached to a 35 mm cylindrical stainless-steel probe (P/35) with the aid of double-sided adhesive tape. The chronic wound surface was mimicked by a gelatin (6.675%) gel substrate equilibrated with 500 µL of simulated wound fluid (SWF). The SWF contained 2% (*w*/*w*) BSA, 0.02 M calcium chloride, 0.8 M sodium chloride in deionized water at pH 7.4. The probe attached to the scaffold approached the model chronic wound surface in tensile mode at a pre-test speed of 0.5 mm/s; contact time of 60 s, trigger force of 0.05 N, an applied force of 1 N followed by detachment at a test speed of 0.5 mm, post-test speed of 1 mm/s, and 10 mm return distance. The adhesive profile of the scaffolds was determined by the peak adhesive force (PAF) required to detach the scaffold from the gelatin surface (stickiness), the total work of adhesion (WOA) signified by the area under the force versus distance curve, and the cohesiveness (the distance traveled by the scaffold until detached) and calculated with the help of the Texture Exponent software (Stable Microsystems Ltd., Surrey, UK).

### 2.5. Exudate Handling Properties

#### 2.5.1. Fluid Intake Evaluation

##### Swelling Studies

The hydration capacity and degree of swelling of the dressings were performed as previously reported [17]. In brief, the lyophilized scaffolds were accurately weighed and immersed in SWF, pH 7.4 at room temperature. The weight change was recorded every 15 min for the first hour and then every hour for 5 h. Before weighing, the hydrated samples were blotted carefully with tissue paper to remove the excess fluid on the surface. The swelling capacity was calculated (*n* = 3) using Equation (1). *I_s_* is the swelling capacity, *W_s_* and *W_i_* are hydrated and the initial weights of the scaffolds, respectively.

(1) $$I_{s}\;(\%)\;=\;\frac{W_{s}-W_{i}}{W_{i}}\;\times \;100$$

##### Porosity

The solvent displacement method was used to investigate the porosity of the lyophilized scaffolds as previously reported [18]. To determine the geometrical dimensions (thickness and diameter) and to calculate the total pore volume of formulations; a digital Vernier caliper was used. Samples (*n* = 3) were weighed (*W*_0_) and immersed in 10 mL of absolute ethanol for 3 h to allow complete saturation, with the void space in the scaffolds displaced by ethanol. Eventually, the samples were carefully removed from the solvent, quickly blotted with tissue paper approximately for 15 s on each side, and immediately weighed (*W_t_*) to avoid the loss of ethanol. Equation (2) was used to calculate the porosity of the dressings.

(2) $$Porosity\;(\%)\;=\;[(W_{t}\;-\;W_{0})/(\rho_{eth}V)\;\times \;100)]$$

(3) $$\rho_{eth}:\;\mathrm{density}\;\mathrm{of}\;\mathrm{ethanol}\;=\;0.789\;g/{cm}^{3}$$

#### 2.5.2. Water Absorption (WA) and Equilibrium Water Content (EWC)

To investigate the maximum water uptake and water holding capacities, the formulations (*n* = 3) were incubated in 10 mL of SWF at 37 °C for 24 h. The initial weight of each scaffold before incubation was recorded whilst the swollen weight was recorded after removing the excess fluid by carefully blotting with tissue paper. The percentage WA and EWC were calculated using Equations (4) and (5), where *W_s_* is the swollen weight and *W_i_* is the initial weight before incubation in SWF.

*WA* (*%*) = [(*W_s_ − W_i_*)/*W_i_*)] × 100 (4)

*EWC* (*%*) = [(*W_s_ − W_i_*)/(*W_S_*)] × 100 (5)

#### 2.5.3. Fluid Loss Assessment

##### Water Vapor Transmission Rate (WVTR)

To determine the moisture permeability of the formulations, the WVTR was measured based on a well-established method [19]. Briefly, the dressing was mounted onto the mouth of a cylindrical tube (16.66 mm diameter) containing 8 mL SWF with an 8 mm air gap between the dressing and the SWF surface. The whole setup was weighed and then placed into a 37 °C incubator for 24 h. The amount of water evaporated per m^2^ per 24 h was calculated using Equation (3). The experiment was performed in triplicate (*n* = 3) for each formulation.

*WVTR* = [(*W_i_ − W_t_*)/A)] × (10^6^) g/m^2^day^−1^) (6)

where *W_i_* is the initial weight of the whole setup, *W_t_* is the weight of the whole setup after incubation for 24 h, and A is the area of the tube (πr^2^).

##### Evaporative Water Loss (EWL)

The formulations previously incubated for WA and EWC experiments were taken out of the SWF and drained and then dried in an oven for 24 h at 37 °C. The weight was noted at regular intervals, and the EWL (*n* = 3) was calculated with Equation (6).

*EWL* (*%*) = [(*W_t_*/*W_i_*)] × 100 (7)

*W_i_* is the initial weight after 24 h incubation in SWF and *W_t_* is the weight after time *t*, respectively.

### 2.6. Protein Leaching

#### 2.6.1. Standard Solution Preparation for Linearity and BSA Content Using HPLC

The BSA was analyzed with an Agilent 1100 series size-exclusive high-performance liquid chromatograph (SE-HPLC) (Agilent Technologies, Cheshire, UK) equipped with an isocratic pump and a UV detector. Calibration series ranging from 10 µg/mL to 200 µg/mL (*r*^2^ > 0.99) from a stock solution of 1 mg/mL BSA was prepared in triplicate using phosphate buffer solution (PBS) pH 7.4. The diluted solutions were filtered (0.45 µm membrane) and eluted through a TOSH TSK-GEL-PW column (7.8 × 300 mm, 10 µm) with a flow rate of 1 mL/min at 22 °C at a detection wavelength of 215 nm and injection volume of 20 µL using PBS (pH 7.4) as mobile phase. The BSA content was determined after hydrating the whole scaffold in 20 mL of SWF (but with no BSA present in the SWF) at 37 °C for 24 h. The concentration was estimated from the equation of the calibration curve.

#### 2.6.2. In Vitro BSA Dissolution Studies

The BSA dissolution studies were performed using a diffusion cell developed in-house containing 20 mL SWF (without BSA) as a dissolution medium. The dissolution medium was filled up to the wire mesh allowing the bottom of the sample to be in touch with the medium. Whole scaffolds (*n* = 3) were placed on the wire mesh and the whole set up placed in an ice bath to prevent protein degradation with constant stirring (500 rpm). At different time intervals, 1 mL of the dissolution medium was withdrawn and replaced with 1 mL of fresh medium. The withdrawn dissolution medium was analyzed for BSA concentration using HPLC. The equation of the calibration curve was used to calculate the concentration of BSA released at each time point up to 72 h.

#### 2.6.3. Evaluation of BSA Release Mechanisms

Different mathematical kinetic models (Equations (8)–(12)) were employed to describe the release kinetics of BSA from the polymeric scaffolds based on dissolution data. Hixson–Crowell (Equation (8)); the cube root law equation describes drug release systems involving a change in surface area and the diameter of particles. The Higuchi drug release model is a time-dependent process that is based on Fick’s law of diffusion and describes the release of drugs from a scaffold as the square root of time (Equation (9)). The zero-order model (Equation (10)) describes a system where the drug release is concentration-independent. In contrast, the first-order rate equation (Equation (11)) describes a concentration-dependent drug release rate. Finally, Korsmeyer–Peppas (or the power law) is useful for the study of drug release from polymeric systems when the release mechanism is not known and involves more than one type of drug release phenomena (Equation (12)) [20,21].

The Hixson–Crowell model:

(8) $$m_{0}^{\frac{1}{3}}-m_{left}^{\frac{1}{3}}=k_{H-C}-c^{t}$$

where *m_t_* is the amount of drug left in the formulation over time *t*, *k_H−C_* is the Hixson–Crowell rate constant.

The Higuchi model:

(9) $$Q_{t}=k_{H\;}t^{0.5}$$

where *Q_t_* is the amount of drug released after time (*t*) and *k_H_* is the Higuchi rate constant

The zero-order kinetic model:

(10) $$Q_{t}-Q_{0}=k_{0}t$$

where *Q_t_* is the amount of drug released in time (*t*), *Q*_0_ is the amount of drug dissolved at time zero, *k*_0_ is the zero-order rate constant.

The first-order kinetic model:

(11) $$\ln\left(Q_{0}-Q_{t}\right)=\ln\left(Q_{0}\right)-k_{1}t$$

where *Q_t_* is the amount of drug remaining at time (*t*), *Q*_0_ is the total amount of drug present initially, *k_t_* is the first-order rate constant.

Korsmeyer–Peppas model:

(12) $$log\;\left(\frac{Q_{t}}{Q_{\infty }}\right)=logk_{k-p}+nlogt$$

where *Q_∞_* is the amount of drug released after an infinite time, *k_k_*_−*p*_ is the Korsmeyer–Peppas rate constant and *n* is the exponent release constant.

### 2.7. MTT (Cell Viability) Assay

To determine the effect of the SA*:COL:HA scaffolds on the viability of human dermal fibroblasts (HDFs) and keratinocytes (KCs), an MTT assay was performed via the indirect contact approach as previously reported [17]. Prior to the MTT testing, the scaffolds were sterilized with UV radiation overnight in a flow cabinet (NU-437-300E, NUAIRE). The sterilized samples were placed in 2.5 mL of complete medium and placed in the incubator (Heracell 150i CO_2_ incubator, Thermo Fisher Scientific, Loughborough, UK) at 37 °C in 5% CO_2_. The supernatant was filtered using 0.2-µm filter, which was necessary, since direct contact of the scaffolds with the complete medium formed gels which affected the cell growth. Cells at a density of 1 × 10^5^ cells/mL were cultured and maintained according to the ATCC-recommended protocol in a 96 well microtiter plate and incubated (37 °C, 5% CO_2_) overnight to allow adherence. The media was removed after 24 h and 100 µL of the sterilized sample was placed into the wells. The plates were incubated for up to 48 h and at each time point (24 h and 48 h) the cells were treated with 10 µL of MTT reagent including the blank (medium only). Thereafter the plates were returned to the incubator for 4 h or more until a purple precipitate was clearly visible. Subsequently, the supernatant was washed and 100 µL was added to all wells including the controls to dissolve the formazan crystals. The plates were further incubated for 30 min and the absorbance was recorded at 492 nm using a micrometer plate reader (Multiscan FC, Thermo Fisher Scientific, Loughborough, UK) equipped with SkanIt for Multiscan FC 3.1. The experiments were carried out in three biological triplicates and repeated three times for each replicate (total *n* = 9) and the percentage of viable cells were calculated using Equation (13). A known toxic compound (Triton-X) was used as the positive control.

(13) $$Cell\;viability\;\left(\%\right)=\frac{At-Ab}{Ac-Ab}*100$$

where *At*, *Ab* and *Ac* are the absorbances of tested samples, media only, and negative control (untreated cells) respectively

### 2.8. In Vitro Blood Clotting Assay

The influence of the prepared scaffolds on blood clotting was performed using whole human blood. Sterile samples were placed in Petri dishes prewarmed at 37 °C for 5 min. Promogran^TM^ (London, UK) collagen-based wound dressing was used as a control for comparison. Human blood was collected from healthy volunteers and anti-coagulated with an acid citrate dextrose tube. 300 µL of blood was instantly dropped over the surface of the scaffold, and the coagulation was triggered by adding 25 µL of 0.2 M CaCl_2_ and incubated for 10 min at 37 °C. Thereafter, the erythrocytes that were not trapped in the clot were hemolyzed with 5 mL of distilled water. The absorbance of the liberated hemoglobin was measured at 542 nm using a spectrophotometer. The absorbance of 300 µL of whole blood diluted in 5 mL of distilled water was used as a reference and assumed to be 100. The clotting study was performed in triplicates (*n* = 3) and clotting index calculated using Equation (14).

(14) $$Blood\;clotting\;index\;\left(BCI\right)=\frac{\left(A\;of\;blood\;in\;contact\;with\;sample\right)}{A\;of\;whole\;blood\;in\;water\;}\times 100$$

*A* = absorbance at 542 nm.

### 2.9. Statistical Analysis

Statistical analysis was performed using one-way ANOVA followed by Sidak’s multiple comparisons tests where appropriate. The differences were considered significant when *p* < 0.05.

## 3. Results and Discussion

### 3.1. Visual Evaluations of Gels and Freeze-Dried Scaffolds

**Figure 1:**
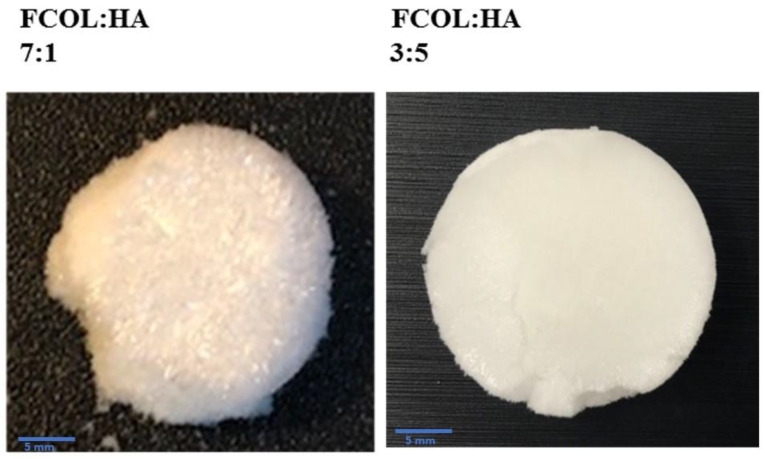
Shows representative digital photographs of blank (non-BSA loaded) composite scaffold prepared from 2% FCOL and HA gels. The gels were generally very thin (of low viscosity) and the resulting scaffolds were fragile, flaky, and very difficult to handle.

**Figure 2:**
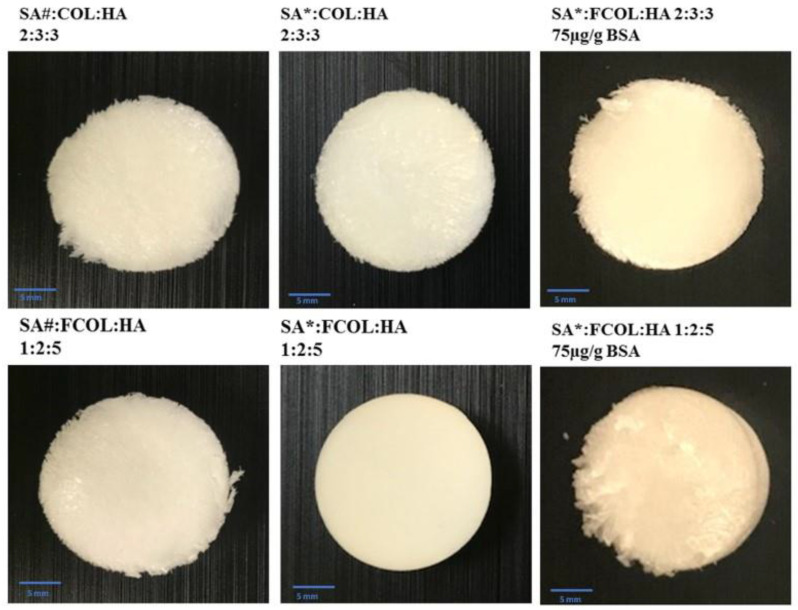
Shows representative images of blank SA:FCOL:HA scaffolds. The incorporation of SA# resulted in flaky edges, in contrast, the SA* produced elegant scaffolds that were easy to handle. Upon BSA loading, these formulations became hard and slightly brittle.

The visual observation of gels was based on: homogeneity upon dispersion in distilled water, presence of aggregates and bubbles, viscosity, and ease of pouring into well plates. Furthermore, the criteria for evaluating scaffolds were softness (flexibility), shrinkage, recovery after application of external force, and ease of removal from well plates. Scaffolds should not be brittle and fragile as hard and brittle scaffolds might be problematic during handling and may further cause damage to the newly formed tissue, while fragile ones will disintegrate during handling or application [18,19]. The FCOL-HA scaffolds were easy to remove from the wells, however, these formulations were also fragile and powdery. Generally, as the proportion of FCOL increased in formulations comprising only two polymers, the scaffolds became more fragile and flakier such as the formulation FCOL:HA 7:1 (Figure 1). Nevertheless, composite scaffolds comprising three polymers (SA:FCOL:HA) using SA with lower G content (SA#) produced clear and homogenous gels that were easy to pour into the well plates. As the concentration of HA increased, the gels became more viscous, while gels with the highest content of FCOL (SA#:FCOL:HA 3:4:1 and 1:2:1) produced fragile and flaky scaffolds. The SA#:FCOL:HA 3:4:1 scaffold was brittle upon removal from the well plate (see Figure 2). When the SA# was replaced with SA containing higher G content (SA*), gels became viscous and upon freeze-drying produced elegant scaffolds that were easy to handle and remove from well plates (Figure 2).

### 3.2. Physico-Chemical Characterization

#### 3.2.1. Scanning Electron Microscopy (SEM)

**Figure 3:**
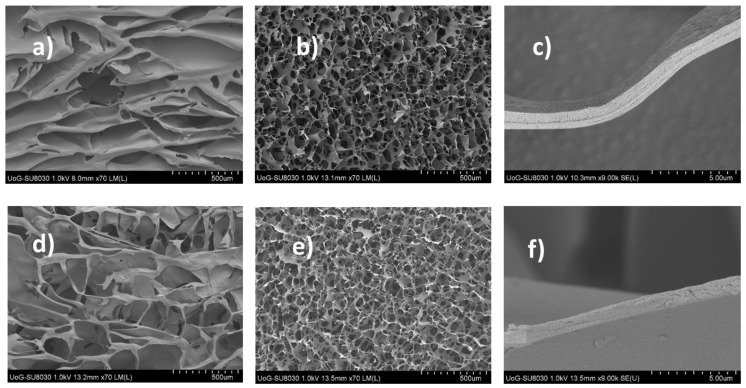
Shows representative topographical images of blank composite SA#:FCOL:HA 1:2:5 scaffolds: (**a**–**c**) top, bottom, and wall thickness respectively showing sheet-like structures with a mean pore size of 440 µm (top) and 107µm (bottom) of the scaffold and wall thickness ranging from 0.442 to 1.864 µm; and (**d**–**f**) SA*:FCOL:HA scaffolds showing interconnected honeycomb-shaped pores with a mean pore size of 282 µm (top) and 127 µm (bottom) and a wall thickness ranging from 0.485 to 0.939 µm.

SEM analysis is key to understanding the morphological architecture of polymers and their behavior within the formulations. The influence of different ratios of SA, FCOL, and HA in blank composite scaffolds, as well as BSA loading on the pore size and wall thickness of the pores, was studied with SEM. The respective micrographs of the various scaffolds are depicted in Figure 3.

The SEM micrographs of the scaffolds containing SA# (i.e., higher M content) demonstrated ribbon-like sheets with a mean pore diameter of 139–440 µm (top of the scaffold) and 81–147 µm (bottom of the scaffold) and wall thickness ranged from 0.44 to 2.50 nm (Figure 3a–c). These differences in size distribution are influenced by the cooling rate during freezing such that a high cooling rate results in small ice crystals and decreases the sublimation rate due to less ice connectivity. In contrast, a slow cooling rate results in large ice crystals with better ice connectivity. The shelf-type freeze dryer used started freezing from the bottom of the gel to the top because the condenser was beneath the shelf. This means that the freezing at the bottom of the gel happened at a higher rate, therefore, resulting in a larger quantity of smaller ice crystals which produced smaller pores upon drying in contrast to the top of the scaffold which produced larger pores [22]. In contrast, SA*:FCOL:HA scaffolds prepared with higher G content SA generally produced porous structures with pores that were smaller in diameter than the scaffolds containing SA#. Furthermore, the pores also became circular shaped and uniform, exhibiting thinner walls (Figure 3d–f)). The formulations containing SA* formed viscous gels and produced three-dimensional continuous phases and interconnected porous networks upon drying. The proposed explanation of this behavior is that SA*-gels with their long G blocks and short elastic segments become a stiff and static network, thus forming a buckled chain [23].

**Figure 4:**
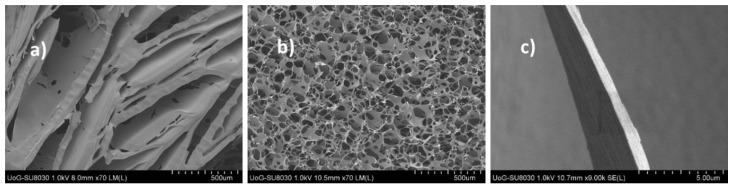
Shows topographical images of the composite SA*:FCOL:HA loaded with 75 µg/g BSA (**a**) top of the scaffold with an average pore size of 275 µm; (**b**) bottom of the scaffold with an average pore size of 122 µm and (**c**) wall thickness ranging from 0.234–0.999 µm.

The differences in pore size and morphology were attributed to the difference in the side chain (G and M) content in the two grades of SA. G-blocks are bent or distorted while M-blocks have extended ribbon-like forms. The M predominant sequence forms an extended ribbon structure due to the dynamic entangled network of the gels, resulting in reduced pores as observed in the micrographs. The BSA-loaded formulations exhibited compact sheet-like porous structures with increased pore size, pore distribution as well as wall thickness. Figure 4 shows the changes in pore size attributed to both BSA and the crystallizing effect of D-mann, which produced more ice crystals in the interior and exterior of the formulation by the formation of hydrogen bonds. Freeze-drying such molecules allows more water molecules to evaporate, resulting in more compact porous structures [24]. These characteristics are expected to have an impact on the ingress of water molecules and exudate handling properties of the resulting scaffolds.

#### 3.2.2. X-ray Diffraction (XRD)

The diffractograms (Figures S1a–d and S2a–c) show a variety of peaks relating to the nature of the pure starting materials and the scaffold formulations. The diffractograms of FCOL showed a broad hallow peak at 2θ of 20.3° indicating its amorphous nature. Additionally, a small peak at 23° 2θ signifies salt impurities that might have been incorporated during the extraction process (Supplementary data Figure S1a). A similar peak for organic and inorganic salt impurities such as sodium chloride (NaCl) and tris hydroxymethyl aminomethane hydrochloride was confirmed by Liu and colleagues [24]. In general, FCOL has low denaturing temperatures and therefore is less stable than mammalian collagen. The lower thermal stability of FCOL depends on the short amino acid (proline and hydroxyproline) residues in contrast to mammalian collagen [25]. A possible explanation for the FCOL diffractogram is the denaturation of FCOL that may have taken place during the extraction process. Due to the loss of crosslinks that are needed to stabilize the collagen fibril structure, the resultant denatured collagen is a random fragmentation of fibrils with no ordered crystalline structure [26]. The diffractogram of SA# (Supplementary data Figure S1b) shows an amorphous structure with peaks at 13.9° and 21.6° and a halo peak at 39.5° 2θ due to the reflection of polyguluronate unit, polymannuronate [27]. From the diffractograms of pure SA, it was also evident that the diffraction peaks representing the polyguluronate content were more intense in SA* than the SA#, which confirms the differences in the G and M content of different SA grades. The diffractogram of HA indicates its amorphous nature. The blending of three polymers changed the original structure of FCOL and SA, resulting in decreased molecular order and therefore increased amorphous nature (Supplementary data Figure S1c).

Moreover, XRD pattern of BSA (Supplementary data Figure S1d) showed two broad diffraction peaks at 9° and 20.3° 2θ signifying the amorphous nature due to the intermolecular H-bonding interactions between the protein chains. However, the diffractograms of BSA-loaded scaffolds (Figure S2c) showed only one small peak at 23° 2θ, which was previously attributed to salt impurities and M content of SA but with lower intensity. The reduced intensity of this peak after BSA loading indicates possible BSA-polymer interaction, and this suggests that the BSA was molecularly dispersed within the polymeric matrix, which is believed to have an impact on the rate of BSA release [28]. Additionally, the incorporation of crystalline D-mann did not alter the amorphous nature of the polymers. According to Pawar and co-workers [29], the physical form (crystalline or amorphous) of polymeric formulations affects many properties such as water uptake, biodegradability, and bioadhesion. They also highlighted that the formulations would have high surface energy due to the less ordered amorphous structures which further helps to improve characteristics such as exudate absorption and prolonged retention of the dressing at the wound site, which will ultimately increase the bioavailability and reduce the need for frequent dressing changes [29].

#### 3.2.3. Attenuated Total Reflectance Fourier Transform Infrared Spectroscopy (ATR-FTIR)

ATR-FTIR spectra, shown in Supplementary data Figure S3a–d shows the characteristic bands of the pure polymers and possible interactions between the components. The characteristic amide I bond of FCOL associated with the vibrations of carbonyl groups along the polypeptide backbone, which represents the α helix or secondary structure was found at 1635 cm^−1^. The amide II functional group was observed at 1521 cm^−1^ mainly from in-plane NH bending and CN stretching vibration. Any changes in amide II represent changes in the secondary structure of proteins such as a decrease in the number of native α helices and an increase in the number of β-sheets [24]. The absorption band between 1241 cm^−1^ and 1411 cm^−1^ (CN stretching and NH bending) was attributed to amide III which confirms the triple helical structure of collagen which shifted to the lower frequency at 1235 cm^−1^, indicating changes in the triple helix structure of FCOL. Furthermore. The amide B band attributed to the CH_2_ asymmetrical stretching was detected at 2929 cm^−1^. The shift to lower frequency (2880 cm^−1^) in the composite formulations is representative of weak interactions [30].

When evaluating the IR spectrum of SA, two spectral regions are usually considered: the first region is between 4000 and 2700 cm^−1^ attributed to O–H and C–H stretching vibrations. The two intense peaks at 1598 and 1409 cm^−1^ (SA*)/1404 cm^−1^ (SA#) were due to asymmetric stretching of the carboxylate group or C–OH deformation vibration (Supplementary data Figure S3b,c). Another strong band at 1024 cm^−1^ (SA#)/1025 cm^−1^ (SA*) was attributed to C–O stretching vibrations. In the fingerprint region, the absorption band around 940 cm^−1^ (SA#)/947 cm^−1^ (SA*) is representative of the C–O stretch of the uronic acid. Lastly, the intensity at 808 cm^−1^ was due to the M residues. In general, the intensity of peaks in SA* was marginally higher than peaks of SA#, especially the peak arising from the uronic residues. This is further confirmation of higher guluronic residues in the SA* [31].

Figure S4a,b shows the possible interactions between the three polymers within the blank (no BSA) composite formulations. A closer examination of the amide peaks showed a notable decrease in the symmetric stretching amide II and amide I peak in the blank composite scaffolds. Amide I red-shifted from 1635 to 1608 cm^−1^ and amide II bands red-shifted from 1521 to 1404 cm^−1^ because of the intramolecular hydrogen bonding between FCOL and other components of the formulation (HA and SA) [32]. The interaction between amine groups of FCOL and carboxylic groups of SA or HA enhances the stability of the gel and provides the matrix for the protein to disperse within the formulation. Another explanation of FCOL interaction with SA and HA is the possible ionic bond formation, i.e., FCOL as an amphiphilic polymer with cationic and anionic groups can interact with SA. In addition, the peak intensities of amide bands were lower in the SA* than the SA# formulations indicating more saturation. Furthermore, in the BSA-loaded formulations; the interaction between SA* and D-mann was confirmed by the shift of the OH-stretch band to a higher wavenumber (1602 cm^−1^) indicating an increase in the strength of hydrogen bond interaction between SA* and D-mann [33].

The incorporation of BSA Figure S4c altered the amide position in the composite scaffolds. The amide I peak observed at 1635 cm^−1^, was shifted to a lower frequency (1599 cm^−1^), which indicates molecular dispersion of the protein within the polymeric matrix [34].

#### 3.2.4. Texture Analysis

##### Hardness

**Figure 5:**
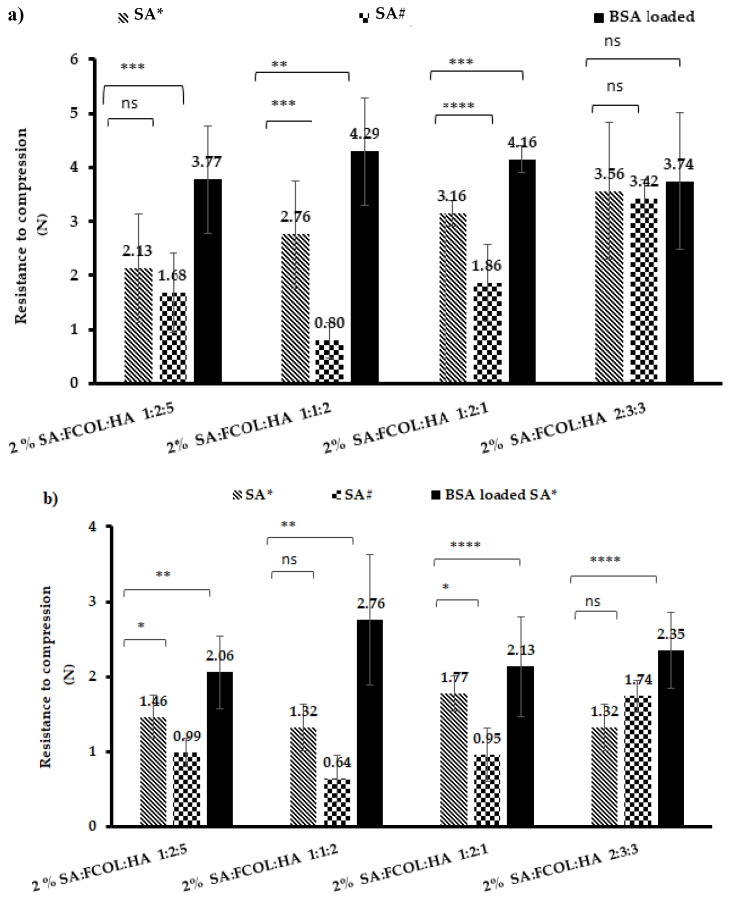
Shows hardness profiles of (**a**) top of the scaffold for composite SA#:FCOL:HA, SA*:FCOL:HA and the optimized BSA loaded formulations (**b**) the bottom of the scaffold for SA#:FCOL:HA, SA*:FCOL:HA and the optimized BSA loaded formulations. Data are shown as mean ± standard deviation (*n* = 3). Statistical differences are shown with *, **, *** or **** denoting *p* < 0.05, *p* < 0.01, *p* < 0.001 or *p* < 0.0001, respectively.

The use of dressings that are too rigid could damage newly formed skin tissue, resulting in prolonged inflammation and wound healing failure. Wound dressings must be bendable, flexible, elastic, and durable and the material chosen must be strong and resistant to applied forces so that it adheres to the wound site [35]. The hardness (peak resistance force to compressive deformation) profiles of the scaffolds are depicted in Figure 5a,b. Incorporating SA# generally resulted in soft scaffolds with average hardness ranging from 0.8–3.42 N (top) and 0.64–1.741 N (bottom). The hardness of the top of the scaffold was significantly (*p* < 0.05) higher than the bottom side of the scaffold (Supplementary data Figure S5). This is because the freeze dryer started freezing from the bottom of the gel upwards such that higher freezing rates at the bottom part produced many small ice crystals becoming more porous upon drying compared to the top part of the scaffold, which produced fewer but larger pores. Consequently, as porosity increases, there is less force required to reach the depth of penetration. Therefore, the bottom part of the scaffold will be ideal for application to the wound bed as it can quickly absorb wound fluid due to the high porosity, and the lower hardness will not cause pain or damage the sensitive newly formed tissue. Although the mechanical stability of the formulations was very low, these formulations were not flaky and brittle as the composite FCOL: HA (2-polymer) scaffolds. The results revealed the suitability of SA as a modifier to enhance the mechanical properties of composite FCOL: HA formulations.

Substituting the SA# with Pronatal LF 10/60 (SA*) resulted in significantly (*p* < 0.05) higher hardness (Figure 5a,b). Therefore, it is evident that the composition of M, G blocks within the SA has significant effects on the mechanical properties. The values obtained are due to the dominant effect of glycosidic bonds between the blocks that are stiff and do not allow alignment of the alginate molecule under compressive force. As observed in the SEM results, SA# produced ribbon-like sheets with larger pores in contrast to SA*, which produced honeycomb-like pores that increased in number but decreased in size. The incorporation of BSA resulted in a less spongy and rigid network giving rise to tighter packing by introducing more inter/intramolecular bonding, thus stiffening the polymeric molecules. As the SEM micrographs revealed, incorporation of BSA resulted in more compact sheet structures, and the difference between mechanical strength of blank and BSA-loaded scaffolds was statistically significant (*p* < 0.05) except for SA*:COL:HA 2:3:3 scaffolds.

##### Adhesion

One unsatisfactory feature of many wound dressings is that they cause damage to the freshly formed tissue upon removal due to their strong adherence to the wound surface. This adhesion is caused by the changes in the properties of exudate which covers the wound surface following an injury. When the exudate is fluid (low viscosity) it causes no problem and due to its surfactant properties, it readily penetrates the fibers of the dressings. However, as it dries, it forms a strong glue binding the dressing into the scab. Removing an adherent dressing before the regenerated epidermis is matured causes pain and damage to the newly formed tissues, which in most cases results in poor patient compliance [36]. On the other hand, a prolonged residence time of the wound dressing is essential to dressing performance since frequently dressing changes are a major cause of patient non-compliance and may also result in complications and delays in wound healing.

**Figure 6:**
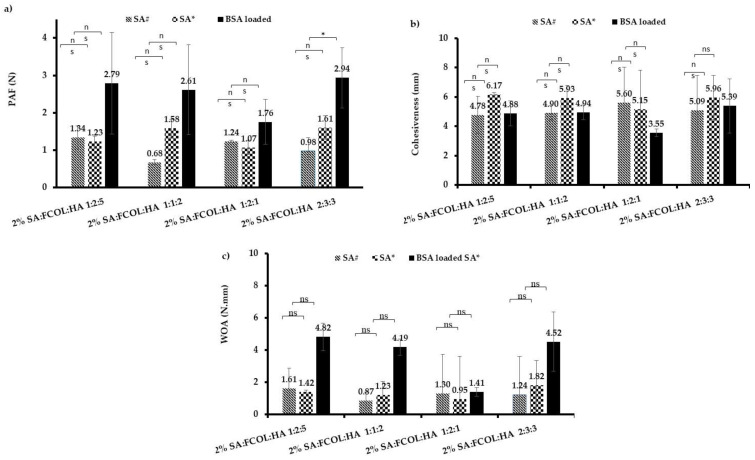
Adhesive profiles of blank 2% SA#:FCOL:HA, blank 2% SA*:FCOL:HA, BSA loaded SA*:FCOL:HA showing: (**a**) peak adhesive force; (**b**) cohesiveness; (**c**) work of adhesion. Data are shown as mean ± standard deviation (*n* = 3). The (*) represents *p* < 0.05 and ns denotes *p* > 0.05.

The in vitro adhesion method estimates the stickiness represented by the PAF required to separate the scaffold from the gelatin substrate after the adhesive bond is established. The WOA represents the amount of energy required to detach the scaffold from the substrate and is calculated from the area under the force vs. distance plot. Cohesiveness determines the ability of the sample to resist separation representing the intermolecular attraction between the substrate and the formulations [18]. Figure 6a–c depicts the adhesive profiles of blank and BSA-loaded formulations. Substituting the SA# with SA* resulted in increased porosity as seen in SEM micrographs of the blank formulations (Figure 3), causing an increase in hydration capacity hence an increase in PAF and WOA. The one-way ANOVA analysis showed a statistically significant difference in the PAF with *p* < 0.05 while the differences in cohesiveness and WOA were not significant (*p* > 0.05).

Alginate is an anionic polymer that exhibits moderate adhesive properties by hydrogen bonding due to the COOH functional groups present in its structure. However, the differences in the adhesive properties of SA* and SA# are not due to differences in H-bonding but could depend on the high affinity of glucuronic acid residues for the calcium ions present in SWF. According to Boateng and co-workers, when alginate comes into contact with an exuding wound, an ion-exchange reaction between the dressing and the ions present in the wound exudate takes place [37].

Selective binding of Ca^2+^ increases with increasing G residues in the chain, whereas the M block has a lower affinity for Ca^2+^. Hecht and colleagues explained that this high ion binding occurs via the formation of “egg-box” regions rich in G residues. The M block dominant SA# has a much flatter structure with more shallow nests for cations to occupy and therefore their selectivity towards Ca^2+^ is lower and only forms complexes at higher concentrations. This possibly explains the higher cohesiveness observed with the SA* formulations [33,34]. However, because the differences were not significant, other factors might be at play, including the ability of SA* to form stronger gels that interacted more strongly with the mucin/gelatin surface, but this will need to be investigated in more detail. Furthermore, the wide variations in both the mechanical hardness and adhesion results between the different scaffolds compared, could be attributed to the small sample numbers (*n* = 3). Future studies using higher sample numbers (*n* = 6 minimum) will help reduce such variability.

The SEM images of protein-loaded dressings (Figure 4) illustrated the disturbance of the pore structure when BSA was loaded into the optimized scaffolds. The reduced porosity possibly resulted in poorer hydration hence decreased PAF and WOA. The ANOVA analysis of the BSA-loaded formulations indicated a significant difference (*p <* 0.05) between the PAF and WOA of the different scaffolds, while the differences in cohesiveness were not significant. The incorporation of BSA and cryoprotectant (D-mann) (Figure 6c), lowered the adhesive profile owing to the increased number of crosslinks that resist rapid interpenetration of water/ion exchange thus resulting in lower adhesive characteristics.

#### 3.2.5. Exudate Handling Properties

##### Fluid Intake Evaluation

- Porosity

Lyophilized scaffolds are typically porous, biocompatible, and biodegradable matrices that serve to provide a suitable environment, such as mechanical support, and physical and biochemical stimuli for optimal cell growth and function. Open porous and interconnected networks are essential for cell nutrition, proliferation, and migration for vascularization and the formation of new tissues. Furthermore, matrices with high porosity enable the effective release of biomolecules such as proteins and provide a suitable substrate for nutrient exchange [35,36]. Although the mean porosity of the blank and BSA-loaded formulations was different (Table 2) the analysis of variance indicated no significant difference (*p* > 0.05) upon substituting SA# with SA* and upon BSA loading. However, the large porosity of sponges achieved by incorporation of SA into the composite FCOL: HA is a promising factor that enables the efficient release of protein from the matrix with the view to attaining an ECM close to the native skin.

- Swelling

**Figure 7:**
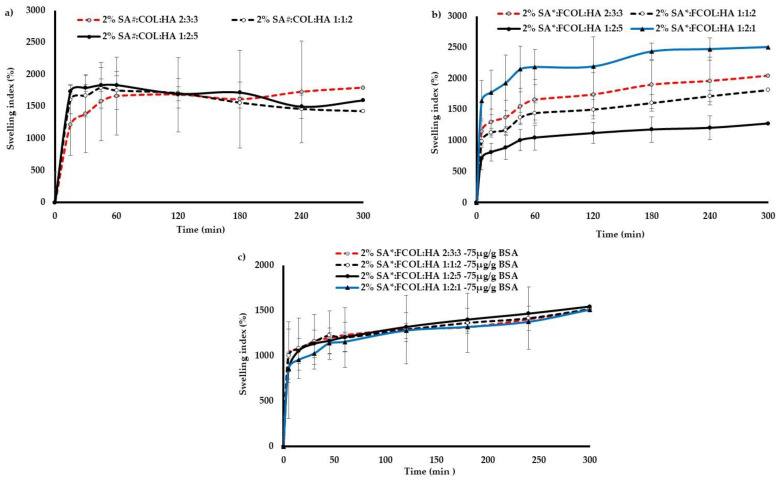
Shows swelling profile of (**a**) SA#, FCOL and HA; (**b**) SA*, FCOL and HA; (**c**) BSA loaded scaffolds in SWF, pH 7.4 (*n* = 3).

Figure 7a–c shows the swelling capacities as a function of time for the various formulations incubated in SWF at pH 7.4 mimicking the wound condition. The scaffolds were able to fully swell, absorbing all the medium added at each time point indicating their good water handling ability.

Swelling is an entropy-driven behavior that increases when water diffuses into the polymeric matrix. When a formulation prepared from hydrophilic polymers such as FCOL, HA, and SA is placed in an aqueous medium, they absorb large quantities of water that causes the polymer chains to move apart, and the entropy increases, leading to swelling. The swelling behavior of the matrix depends on polymer structure, the nature of the swelling medium, and the degree of crosslinking of polymers. Further, swelling behavior also determines the composite matrix properties [38] and is an important characteristic in controlled drug delivery systems since it affects the rate of solvent ingress into the matrix and the drug diffusion through the gel layer of the matrix [39].

Generally, the swelling capacity of all formulations containing SA# increased rapidly within the initial 15 min, followed by a steady increase for 5 h. The formulations such as SA#:FCOL:HA 1:2:1 and SA#:FCOL:HA 3:4:1 disintegrated immediately upon immersion in SWF and were therefore eliminated at this stage of optimization. The remaining formulations were also not capable of maintaining their structural integrity after 5 h hydration. Although replacing the SA# with SA* did not indicate any statistically significant difference (*p* > 0.05) in swelling, the formulations containing SA* were structurally more stable. The SA* based formulations (Figure 7b) also showed an immediate increase in the first 15 min and steadily increased until 5 h. The fast-initial swelling was mainly caused by the porous structure of the scaffolds, which allows the SWF to infiltrate the porous matrix rapidly, while the subsequent steady increase in swelling was attributed to the water-binding capacity of SA, FCOL, and HA. It was evident that the incorporation of SA* contributed to a steady and slower rate of swelling of the formulations, thus enhancing the mechanical stability of the components. This indicates that the chemical structure of SA has important implications for the functional clinical performance of the dressing. The SA# containing scaffolds can be easily washed off from the wound surface after being moistened by the exudate compared to dressings containing SA*. The latter could swell in the presence of wound fluid and maintain its gel structure after an extended period owing to its interaction with the Ca^2+^ ions in the SWF [40].

It is interesting to note that for scaffolds containing a higher concentration of HA, a lower swelling capacity was related to a lower (%) porosity, lower (EWC), and (WA) (see Table 2). In contrast, the high swelling capacity of formulations with a higher content of FCOL exhibited higher (%) porosity, (EWC) and (WA) indicating the weakness of FCOL in the hydrated state because of low crosslinking. The high swelling ability of the formulations was attributed to the hydrophilic functional groups such as NH_2_ and COO- present in the biopolymers [41]. The difference in the swelling capacity of blank and BSA-loaded formulations was statistically significant (*p* < 0.05). As a result of a higher degree of crosslinking between the BSA, polymers, and cryo-protectant, the water handling properties of the BSA-loaded formulations decreased (see Figure 7). This is due to the fact that BSA leads to more considerable interaction and shrinkage of the cavities and pores leading to fewer void spaces available for the ingress of SWF, thus decreasing the swelling capacity [28]. The excellent water uptake capacity is an essential factor for the wound healing activity of biological wound dressing as fluid uptake is required to maintain a moist environment over the wound bed.

- Water Absorption (WA) and Equilibrium Water Content (EWC)

The water absorption (WA) and equilibrium water content (EWC) of wound dressings are important factors that assess the capacity of formulation to quickly absorb and retain exudate. As presented in Table 2 the WA and EWC of the composite blank scaffolds ranged from 696 ± 267 to 1382 ± 20% and 86 ± 5 to 94 ± 0.5% respectively. These results indicated high water absorption for formulations containing higher FCOL in contrast to higher HA-containing formulations. Although HA can swell up to 1000 times its original weight, it was not able to reach its maximum hydration capacity and this is because solute salts present in the SWF retard the water ingress, hence reducing binding sites available for hydrogen bonding [42]. Furthermore, WA can be related to the pore size, structure, and interconnectivity. Due to low mechanical stability and sheet-like structure, FCOL allows faster water absorption, whereas the high HA-containing formulation absorbed water more slowly, due to its compact pore structure.

The difference in WA and EWC between SA#, SA* and BSA loaded were statistically significant (*p* < 0.05) implying an increase in crosslinking upon BSA loading that further decreased the pore diameter, subsequently resulting in decreased WA. The ability to absorb and retain large quantities of exudate extends the physical integrity of the dressing and reduces the frequency of dressing change while sustaining a moist environment for wound healing. Therefore, it can be concluded that the formulations developed were suitable to be used as absorbent dressings.

##### Fluid Loss Assessment

- Water Vapor Transmission Rate (WVTR)

Wound healing is a complicated process that requires a desirable microenvironment with moisture content being one of the most critical factors because cells in the wound thrive in a moist microenvironment. For patients suffering from severe acute or chronic wounds, the fluid conserving function of healthy skin is destroyed, and the fluid loss is approximately twenty times greater than that of healthy skin. If the wound is directly exposed to air, it dehydrates, and a scab is formed. Although the scab aims to protect the wound from bacterial invasion, the cells in the dehydrated state lose function and die, whereas moisture accelerates wound healing [43]. According to Ahmed and Boateng [17], the WVTR indicates the ability of a dressing to absorb fluid and draw it out from the wound across the dressing to the atmosphere which is correlated to the porosity and thickness of the dressing. Similarly, in this study, formulations with high porosity such as SA#:FCOL:HA; 2:3:3 and 1:2:5 exhibited high WVTR of 2354 ± 67 and 2328 ± 22 g/m^2^/day respectively, followed by SA#:FCOL:HA 1:2:1 (2340 ± 35) and SA#:FCOL:HA 1:1:2 (2333 ± 63) g/m^2^/day (Table 2). Replacing the SA# with SA* did not affect the WVTR; however, when BSA was loaded, the WVTR increased significantly (*p* < 0.05).

As previously reported by Álvarez-Suárez and colleagues [44] the amount of exudate produced by a patient with a burn wound is ~5000 g/m^2^/day, while exudate production for chronic ulcers ranges from 4000 to 12,000 g/m^2^/day. Therefore, formulations in this study with WVTR ranging from 2527 ± 38 to 2405 ± 69 are capable of transmitting about 50% exudate from burn and mild exuding chronic wound bed [16,45]. This implies that the composite SA:FCOL:HA scaffolds will be able to provide a moist environment to enable the progression of wound healing, whilst preventing the collection of excess exudate, which could cause maceration of healthy skin around the wound as well as prevent the possibility of infection developing.

In a comparative study of spongy scaffolds prepared using collagen from fish and porcine sources conducted by Shi and colleagues [46], higher WVTR was achieved with FCOL that was able to maintain optimal moisture, resulting in scar-free healing in rabbit models. Comparatively in this study, the BSA-loaded composite SA*:COL:HA formulations attained higher WVTR than SA#:COL:HA but lower than commercial Algisite Ag^®^ wound dressing as previously reported [17]. This suggests that our findings could be helpful in designing wound dressing with the ability to produce a moist wound healing environment for the proliferation of epidermal cells and fibroblasts.

- Evaporative Water Loss (EWL)

One of the most critical properties of biological moist wound dressing is its ability to control the EWL from the wound site since excessive water loss may lead to difficulty in regulating temperature, dehydration, and healing. On the other hand, exudate build-up may lead to pain, leakage, bacterial infiltration, and maceration of healthy tissues. Therefore, it is essential for wound dressings to maintain an intermediate level of fluid retention. Wound covering is the first line of defense for maintaining body fluid in balance hence the quantification of the EWL would improve the estimation of the body’s water loss and help choose appropriate treatment procedures [47].

**Figure 8:**
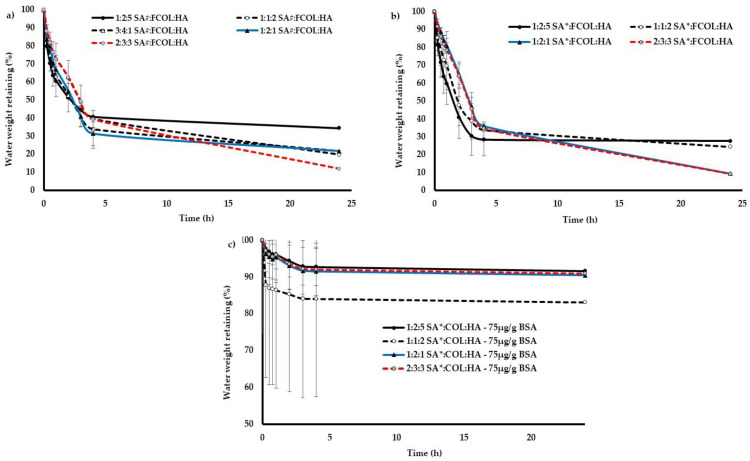
EWL from (**a**) SA#:FCOL:HA (**b**) SA*:FCOL:HA and (**c**) BSA loaded SA*:FCOL:HA formulations.

As seen in (Figure 8a) the SA# scaffolds generally showed 30-40% water loss within 1 h which was similar to that of commercially available Algisite Ag^®^ dressing previously tested by Ahmed and Boateng [17] under similar experimental conditions. This increased to 61–69% within 4 h. Conversely, the SA* formulations mostly showed 22–30% water loss in 1 h and increased to 72–76% after 4 h (Figure 8b). Generally, the difference in EWL upon substitution of SA# with SA* was statistically not significant (*p* > 0.05). The results reveal that these materials will lose most of their water content in a short time when exposed to air in dry conditions, thus enabling the gels to absorb more exudate from the exuding wounds.

A significant decrease (*p* < 0.05) in EWL was observed when BSA was incorporated into optimized formulations (Figure 8c). The formulations retained 83% to 90% of water, which may be due to the water sorption and retention properties of BSA and its interaction with the excipients. The composite formulations became denser with increased concentrations of BSA, resulting in the presence of micro and nano-sized pores and reducing the water evaporation thereby increasing water retention.

#### 3.2.6. Protein Leaching

**Figure 9:**
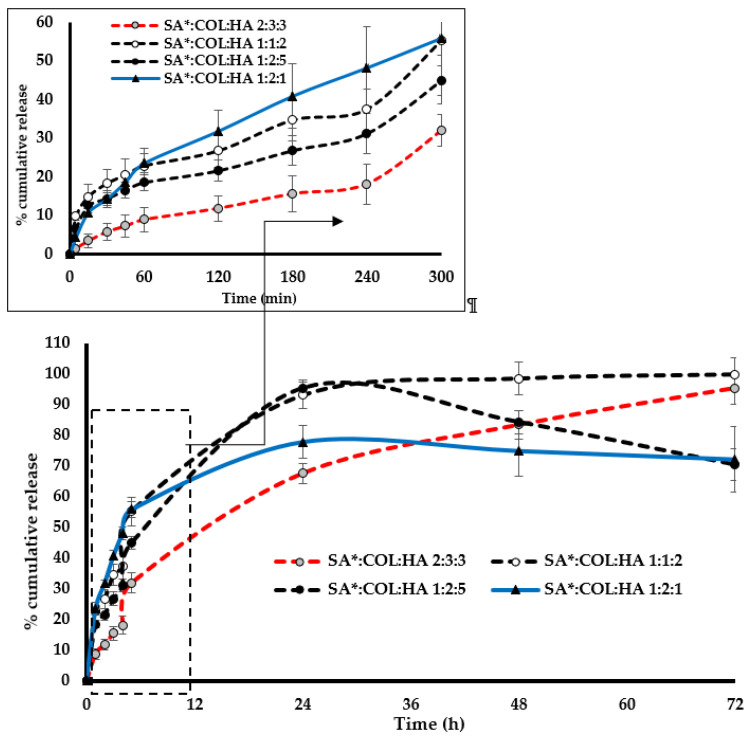
Percentage cumulative protein release profiles of 75 µg/g BSA loaded SA*:FCOL:HA scaffolds (*n* = 3 ± SD).

The percentage cumulative release profile of BSA from composite 2% SA*:FCOL:HA scaffolds for the first 5 h is shown in (Figure 9). The release profile can be divided into two phases: an initial near-linear release phase and a relatively steady phase. Slow hydration of the scaffolds was observed in the first 60 min, with about 8–23% of the protein released within this time (Figure 9-inset). The formulation with a higher HA content (SA*:FCOL:HA 2:3:3 and SA*:FCOL:HA 1:2:5) swelled at a slower rate compared to those containing higher amounts of FCOL (SA*:FCOL:HA 1:2:1). About 30 to 55% of BSA was released from the dressings within 5 h followed by a steady release of the remaining BSA in 72 h. To predict the release mechanisms of BSA from the dressings, the results were fitted to mathematical models as depicted in Table 3. The BSA release mechanism was confirmed by Korsmeyer–Peppas plots with R^2^ values ranging from 0.896 to 0.971 and slope (*n*) < 0.5 indicating that the BSA release mechanism was governed by quasi-Fickian diffusion [48]. According to this model, as liquid penetrates the matrix the polymer chains undergo swelling and become disentangled, which is dependent on the solvent properties and the crosslinks within the matrix. Therefore, the hydration enhances the inter-diffusion process that dissolves the BSA and allows its release through a process controlled by diffusion.

A diffusion coefficient *n* ≤ 0.5 indicates that the release is controlled by Fickian diffusion which is associated with the concentration gradient, diffusion distance, and the degree of swelling [39]. However, the Higuchi model was proven to be suitable to explain the release mechanism of BSA from the SA*:COL:HA 1:1:2 scaffold, which indicates that the release of BSA from the encapsulating matrix into the release medium was unidirectional [49].

### 3.3. MTT Assay

**Figure 10:**
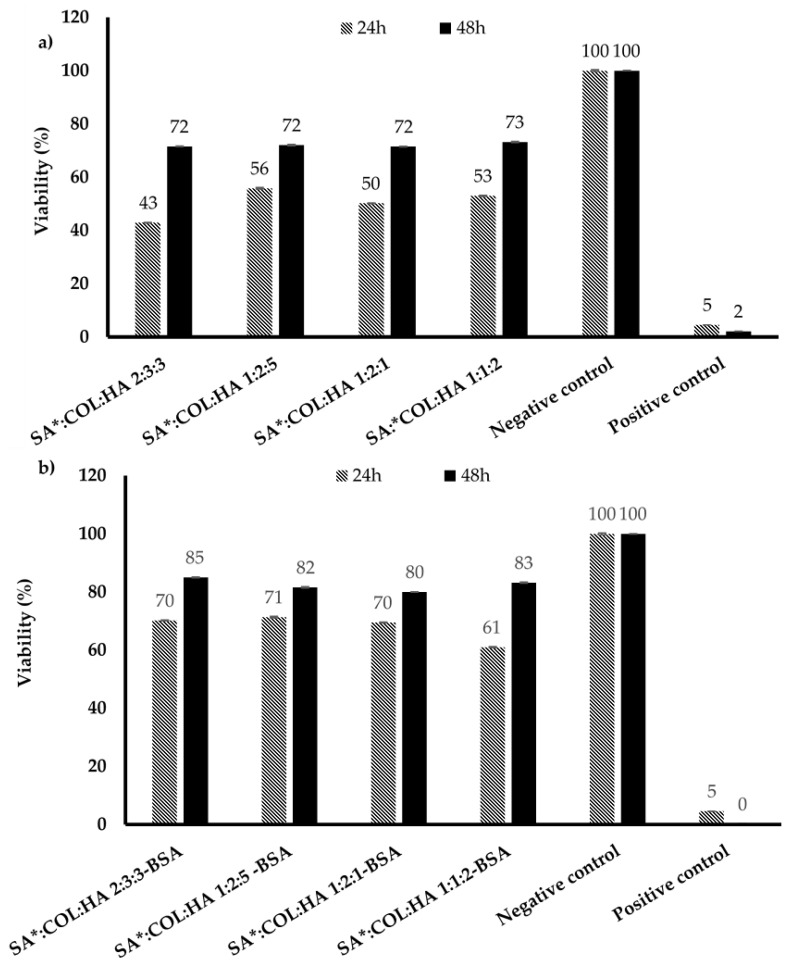
Cell viability of (**a**) primary KCs after exposure to the SA*COL:HA blank scaffolds for 24 and 48 h (mean ± SD *n* = 9); (**b**) primary KCs after exposure to the BSA loaded SA*COL:HA scaffolds for 24 and 48 h (mean ± SD, *n* = 9).

**Figure 11:**
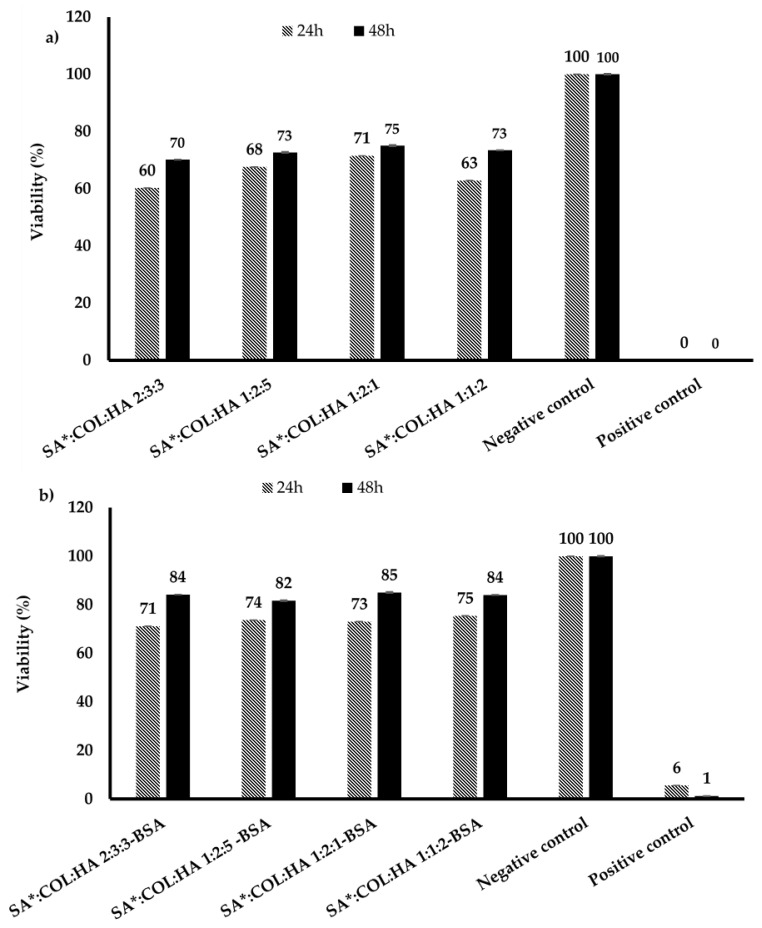
Cell viability of (**a**) primary HDFs after exposure to the SA*COL:HA blank scaffolds for 24 and 48 h (mean ± SD, *n* = 9); (**b**) primary HDFs after exposure to the BSA SA*COL:HA loaded scaffolds for 24 and 48 h (mean ± SD, *n* = 9).

Cytotoxicity is one of the most important factors that determine the biocompatibility and safety of biomaterials. This is an essential requirement recommended by the international organization for standardization (ISO) for the safe use of medical devices and materials. The biocompatibility of the BSA loaded scaffolds was evaluated utilizing HDF and KCs (the major cellular components of the dermis and epidermis and having several important roles in the wound healing process) after incubation with the SA*:COL:HA scaffolds for 24 and 48 h) and the results are shown in Figure 10 and Figure 11. The scaffolds had satisfactory cytocompatibility with HDFs and KC after 48 h, implying minimal inhibition of cell proliferation compared to the known toxic positive control. However, all the scaffolds showed lower cell viability values than the commercial control samples at both time points. In addition, the results indicate a time dependent impact of the scaffolds on the cell viability and proliferation with lower percentages after 24 h (especially for the blank formulations) but increased to the accepted levels after 48 h. This is a limitation that will need more investigations in future studies. Further, incorporation of BSA seems to increase the cell viability of the scaffolds (see Figure 10b and Figure 11b) by modifying their interaction with cells, reducing the chance of cellular uptake thus activating growth factors necessary for their proliferation [50].

### 3.4. Whole Blood Clotting

**Figure 12:**
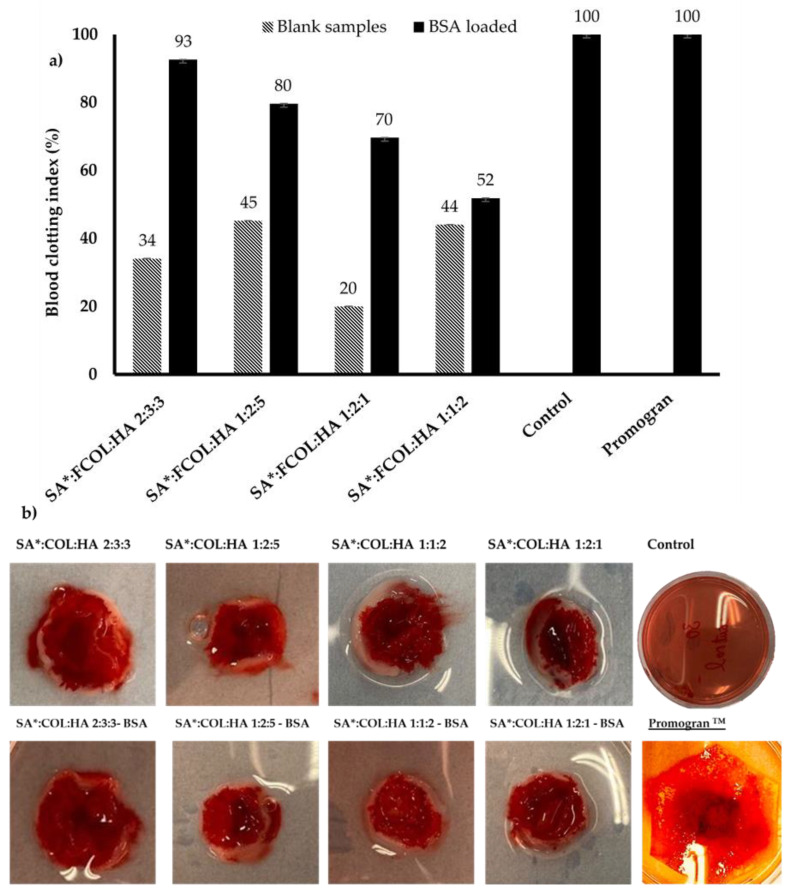
(**a**) The blood clotting index (BCI) of the scaffolds after being in contact with whole human blood; (**b**) photographic images of the scaffolds after incubation.

Blood compatibility is one of the important properties required in wound dressings. Whole blood clotting measurements were performed to investigate the coagulation effect of SA*:FCOL:HA compared to Promogran^TM^, a commercially available collagen-based wound dressing for the treatment of chronic wounds. During blood clot formation, the quantity of the entrapped hemoglobin by the fibrin increases while at same time the amount of free hemoglobin decreases. Therefore, this could be used as an indicator to evaluate the potential of a dressing to induce clotting. Figure 12a demonstrates BCI of the scaffolds. The BCI of Promogran^TM^ was 100% after 10 min of incubation higher than the BCI of SA*:FCOL:HA scaffolds, indicating that SA*:FCOL:HA scaffolds significantly increased the blood clotting rates compared to Promogran^TM^ as seen in the photographic images shown in Figure 12b due to the hemostatic activity of the three biopolymers SA*, FCOL, and HA. The SA is ionic after coming into contact with blood and initiates an ion exchange process between its sodium ions and the calcium ions present in the blood, which activates the platelets and thereby enhances the blood coagulation activity. Collagen plays a pivotal role in hemostasis and provides sites for platelet adhesion, activation and accumulation in the wound area thus initiating the coagulation cascade which sustains the hemostasis process. In addition, due to its excellent water retention and swelling properties, HA maintains a favorable environment for hemostasis, thus increasing cell adhesion and cell migration [51]. The scaffolds loaded with model protein BSA significantly decreased the coagulation, which can be attributed to the anticoagulant action of BSA due to its inhibitory effects on platelet aggregation [52].

## 4. Conclusions

The composite scaffolds containing SA with low G content (SA#:FCOL:HA) were mostly brittle and flaky and hard to handle and produced ribbon-like structures at the microscopic level. The pore size and compositions of these formulations further affected their mechanical and functional properties. Substituting SA# with SA* resulted in flexible, soft, and non-brittle scaffolds and generally produced porous structures with a continuous phase and interconnected network and allowed these formulations to swell for a more extended period. The XRD results showed that incorporation of BSA reduced the molecular order and therefore increased the amorphous nature of the dressings, which suggests that the model protein (BSA) was molecularly dispersed within the matrix. Scaffolds prepared from 2% gels containing SA with high G content (SA*:FCOL:HA), showed desirable characteristics required for wound dressing with a hardness range of (3.7–4.3 N) that can withstand normal stresses but is flexible to prevent damage to newly formed tissues. The porosity of the BSA-loaded scaffolds was above 88%, WA and EWC ranged from 380–493% and 79–82%, respectively, while the incorporation of BSA reduced the EWL. The composite SA*:FCOL:HA scaffolds exhibited a two-phase release profile, a linear phase during the first hour followed by a steady release for up to 72 h. This was deemed relatively fast compared to commercially available dressings and therefore requires further modification. MTT assay showed that the scaffolds did not inhibit proliferation of HDFs and KCs, therefore biocompatible. In vitro blood analysis performed on human whole blood confirmed that the BSA-loaded SA*:COL:HA scaffolds reduced the blood clotting index (BCI) by up to 20% compared to a commercially available sponge. These features confirm that SA*:COL:HA scaffolds could be applied as multifunctional wound dressing for potential chronic wound healing.

## Figures and Tables

**Table 1:** Composition of biopolymers used to prepare 2% composite FCOL:HA and 2%. SA:FCOL:HA gels including D-mannitol as a cryoprotectant at 20% of total polymer weight for BSA-loaded gels.

Formulations	Constituent Amount (mg)		
	SA#/SA*	FCOL	HA
2-polymer composite gels			
FCOL:HA 7:1	0	1750	250
FCOL:HA 3:1	0	1500	500
FCOL:HA 1:1	0	1000	1000
FCOL:HA 5:3	0	1250	750
FCOL:HA 3:5	0	750	1250
3-polymer composite gels			
SA:FCOL:HA 3:4:1	750	1000	250
SA: FCOL: HA 1:2:1	500	1000	500
SA: FCOL: HA 1:1:2	500	500	1000
SA: FCOL: HA 2:3:3	500	750	750
SA: FCOL: HA 1:2:5	250	500	1250

SA* represents SA Protanal 10/60 that has G/M % ratio of 70/30 (low G content); SA# represents SA with G/M % ratio 39/61 (high G content).

**Table 2:** Shows the exudate handling properties of blank and BSA-loaded formulations (*n* = 3 ± SD).

Sample	Low G Content SA (SA#)				High G Content SA (SA*)			
	Porosity (%) ± (SD)	EWC (%) ± (SD)	WA (%) ± (SD)	WVTR (g/m^2^ day ^−1^) ± (SD)	Porosity (%) ± (SD)	EWC (%) ± (SD)	WA (%) ± (SD)	WVTR (g/m^2^ day ^−1^) ± (SD)
2% SA:FCOL:HA 1:2:5	79 ± 2	86 ± 5	696 ± 267	2328 ± 22	83 ± 2	88 ± 2	843 ± 210	2429 ± 53
2% SA:FCOL:HA 1:2:1	76 ± 6	89 ± 4	948 ± 382	2340 ± 35	94 ± 16	94 ± 1	1382 ± 20	2183 ± 45
2% SA:FCOL:HA 2:3:3	70 ± 4	90 ± 2	956 ± 266	2354 ± 67	94 ± 14	93 ± 1	1252 ± 56	2255 ± 41
2% SA:FCOL:HA 1:1:2	77 ± 3	91 ± 2	1015 ± 287	2333 ± 63	96 ± 9	88 ± 2	793 ± 132	2393 ± 38
2% SA:FCOL:HA 3:4:1	76 ± 3	92 ± 1	1243 ± 299	2368 ± 38	-	-	-	-
2% SA:FCOL:HA 2:3:3 75 µg/g BSA	-	-	-	-	88 ± 4	80 ± 1	405 ± 46	2527 ± 38
2% SA:FCOL:HA 1:2:5 75 µg/g BSA	-	-	-	-	88 ± 5	79 ± 1	380 ± 14	2488 ± 14
2% SA:FCOL:HA 1:2:1 75 µg/g BSA	-	-	-	-	89 ± 3	80 ± 1	404 ± 34	2475 ± 78
2% SA:FCOL:HA 1:1:2 75 µg/g BSA	-	-	-	-	91 ± 7	82 ± 5	493 ± 213	2405 ± 69

**Table 3:** BSA release data fitted to various kinetic models.

	Higuchi		Korsmeyer–Peppas			Zero Order		First Order	
	K_H_	R^2^	K_K-P_	*n*	R^2^	K_0_	R^2^	K_1_	R^2^
SA*:FCOL:HA 2:3:3	1.559	0.890	0.121	0.596	0.971	0.023	0.897	0.001	0.067
SA*:FCOL:HA 1:1:2	2.615	0.941	0.447	0.478	0.897	0.094	0.795	0.001	0.036
SA*:FCOL:HA 1:2:5	1.318	0.798	0.366	0.476	0.913	0.018	0.602	0.001	0.052
SA*:FCOL:HA 1:2:1	1.162	0.772	0.359	0.495	0.896	0.016	0.571	0.001	0.023

## Supplementary Materials

The following supporting information can be downloaded at: https://www.mdpi.com/article/10.3390/polym14081550/s1, Table S1: shows FTIR bands for FCOL, HA, SA and BSA with peak characteristic and assignments. Bands showing shifts within the formulation dressings are highlighted in bold font and their original peaks shown in the right column, Figure S1: shows diffractograms for (a) pure FCOL; (b) pure SA# and SA*; (c) pure HA (d) pure BSA powder, Figure S2: shows diffractograms for (a) composite SA#:FCOL:HA; (b) composite SA*:FCOL:HA; (c) BSA loaded composite scaffolds, Figure S3: shows FTIR spectra of (a) pure FCOL; (b) pure SA#; (c) pure SA*; (d) BSA powder, Figure S4: shows FTIR spectra of (a) composite SA#:FCOL:HA; (b) SA*: FCOL:HA where the uronic acid residue with higher G content has marginally higher intensity. The amide I and amide II bands appeared to shift toward lower wavenumbers, i.e., red shift (from 1635 to 1608 cm^−1^ amide I), (1521 to 1404 cm^−1^ amide II); (c) BSA loaded formulations showed further shifts of amide bands, indicating the dispersion of the protein within the polymeric matrix. All the band shifts observed for the composite formulations are shown in Table S1 of supplementary data, Figure S5: shows the hardness profiles of top and bottom of the formulated scaffolds of 2% SA#:COL:HA, SA*:COL:HA and BSA loaded formulations (*n* = 3). The difference in hardness between the top and bottom of the various scaffolds was significant with *p* < 0.0001 at 95% confidence interval.
