# Supplementary material for: Composite Fish Collagen-Hyaluronate Based Lyophilized Scaffolds Modified with Sodium Alginate for Potential Treatment of Chronic Wounds
Source: Polymers (Basel). 2022 Apr 11;14(8):1550. doi: 10.3390/polym14081550 (PMC9031246; doi:10.3390/polym14081550)
Supplement: Supplementary file 1 [file polymers-14-01550-s001.zip › polymers-1652881-supplementary.pdf]

Supplementary Information

Composite Fish Collagen-Hyaluronate Based Lyophilized Scaffolds Modified with Sodium Alginate for Potential Treatment of Chronic Wounds

Meena Afzali and Joshua Siaw Boateng *

School of Science, Faculty of Engineering and Science, University of Greenwich, Kent ME4 4TB, UK; m.afzali@greenwich.ac.uk

* Correspondence: j.s.boateng@gre.ac.uk or joshboat40@gmail.com; Tel.: +44-2083318980

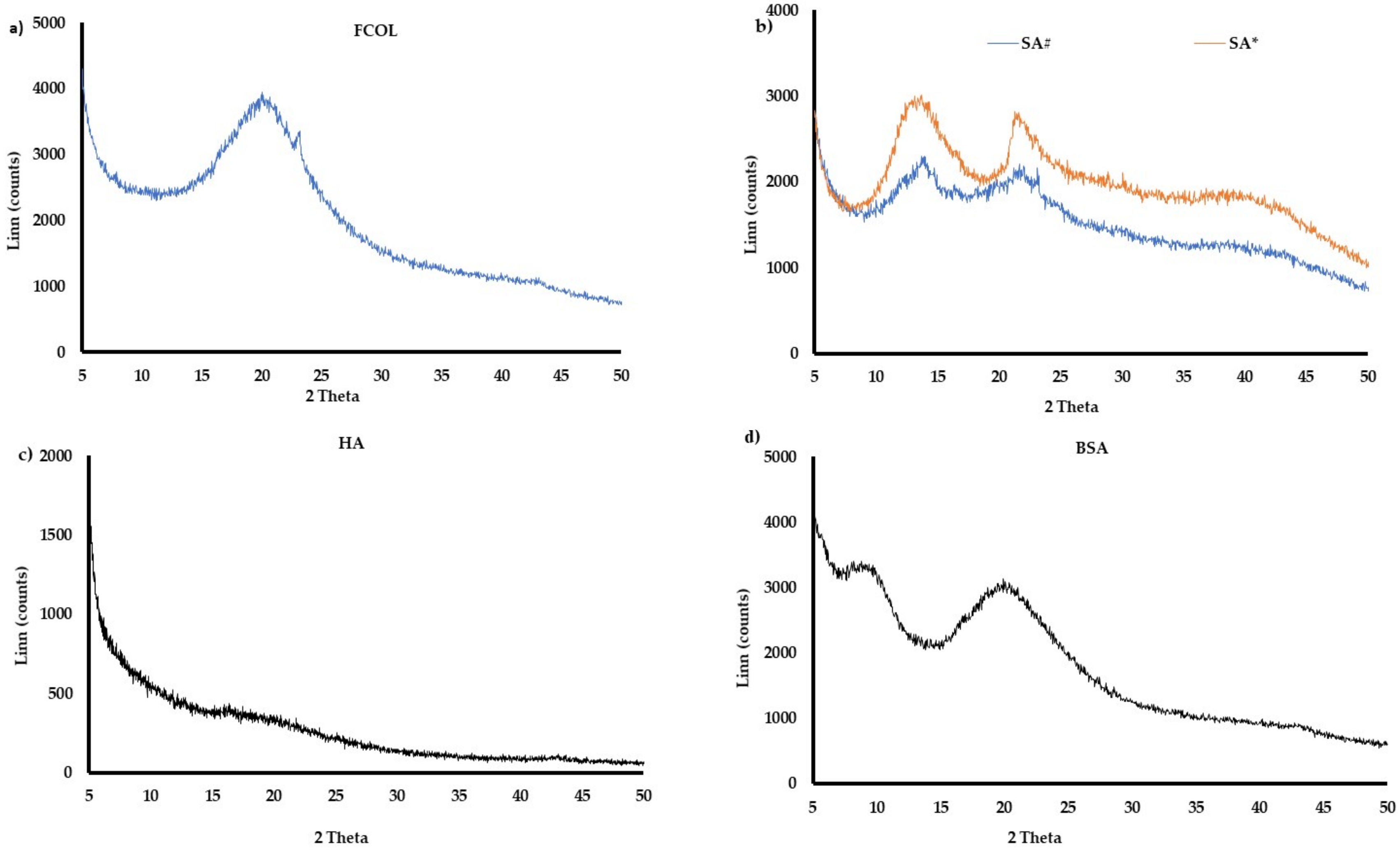

Figure S1. Shows diffractograms for (**a**) pure FCOL; (**b**) pure SA# and SA*; (**c**) pure HA; (**d**) pure BSA powder.

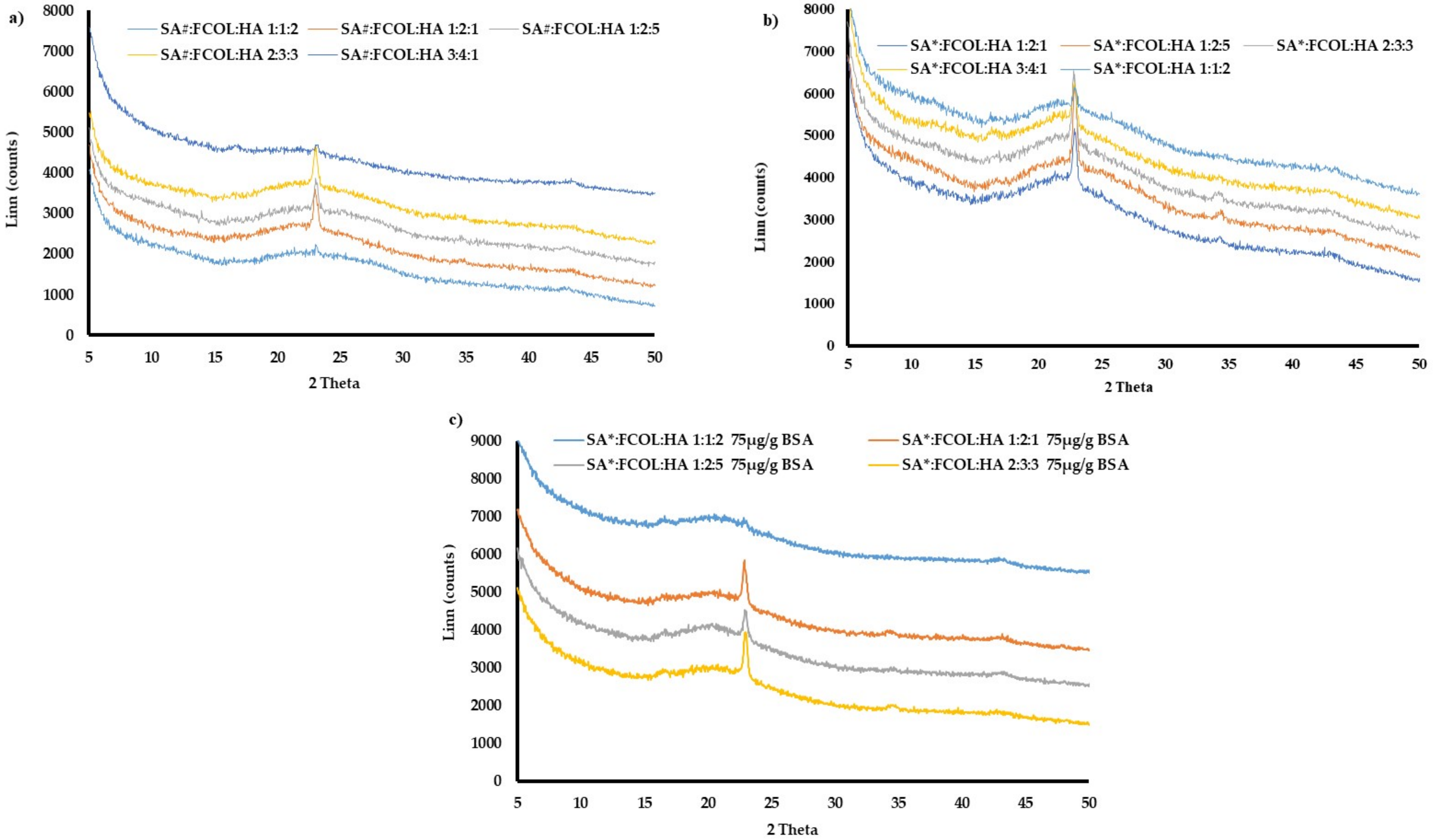

Figure S2. Shows diffractograms for (**a**) composite SA#:FCOL:HA; (**b**) composite SA*:FCOL:HA and (**c**) BSA loaded composite scaffolds.

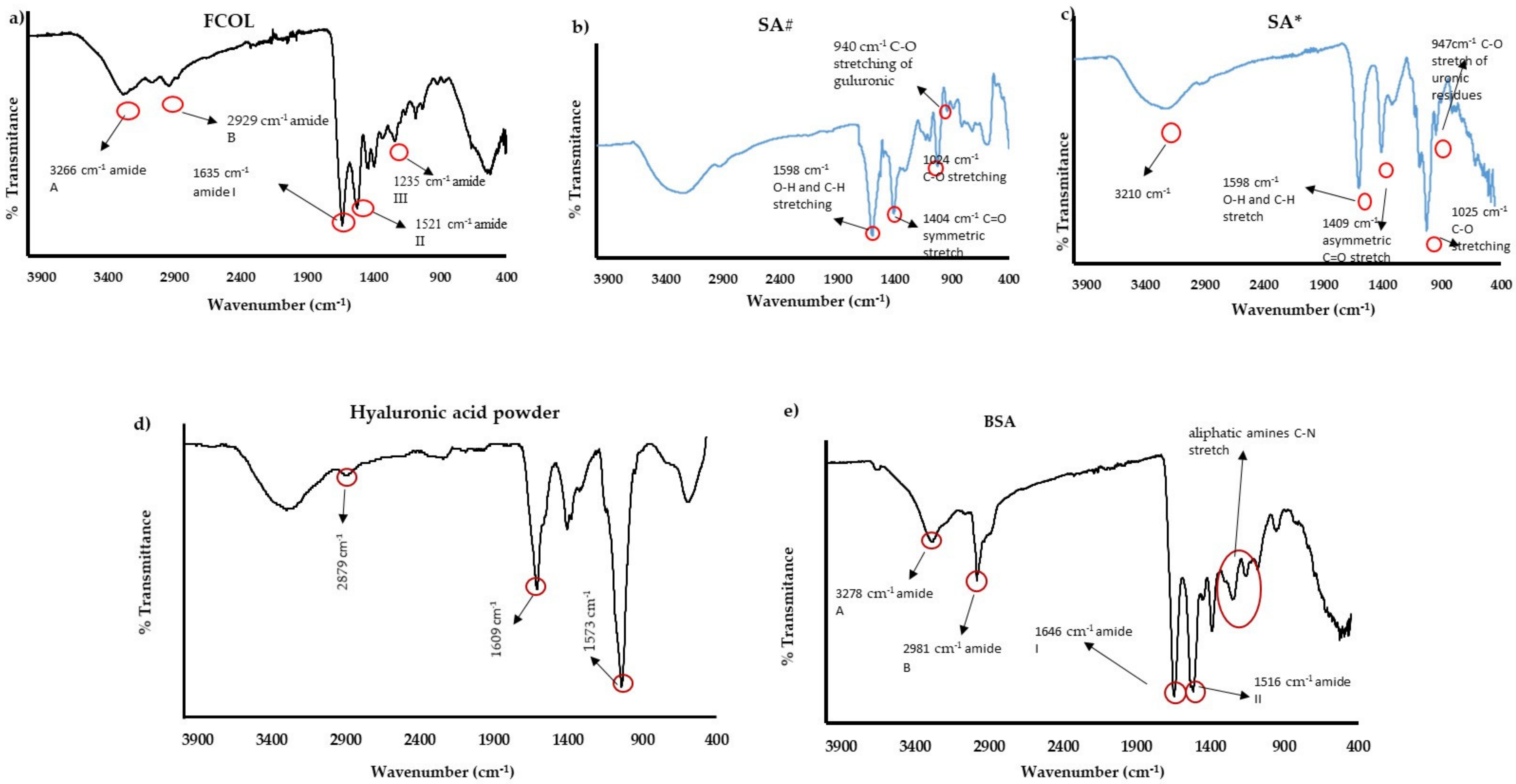

Figure S3. Shows FTIR spectra of (**a**) pure FCOL; (**b**) SA#; (**c**) Pure SA*; (**d**) BSA powder.

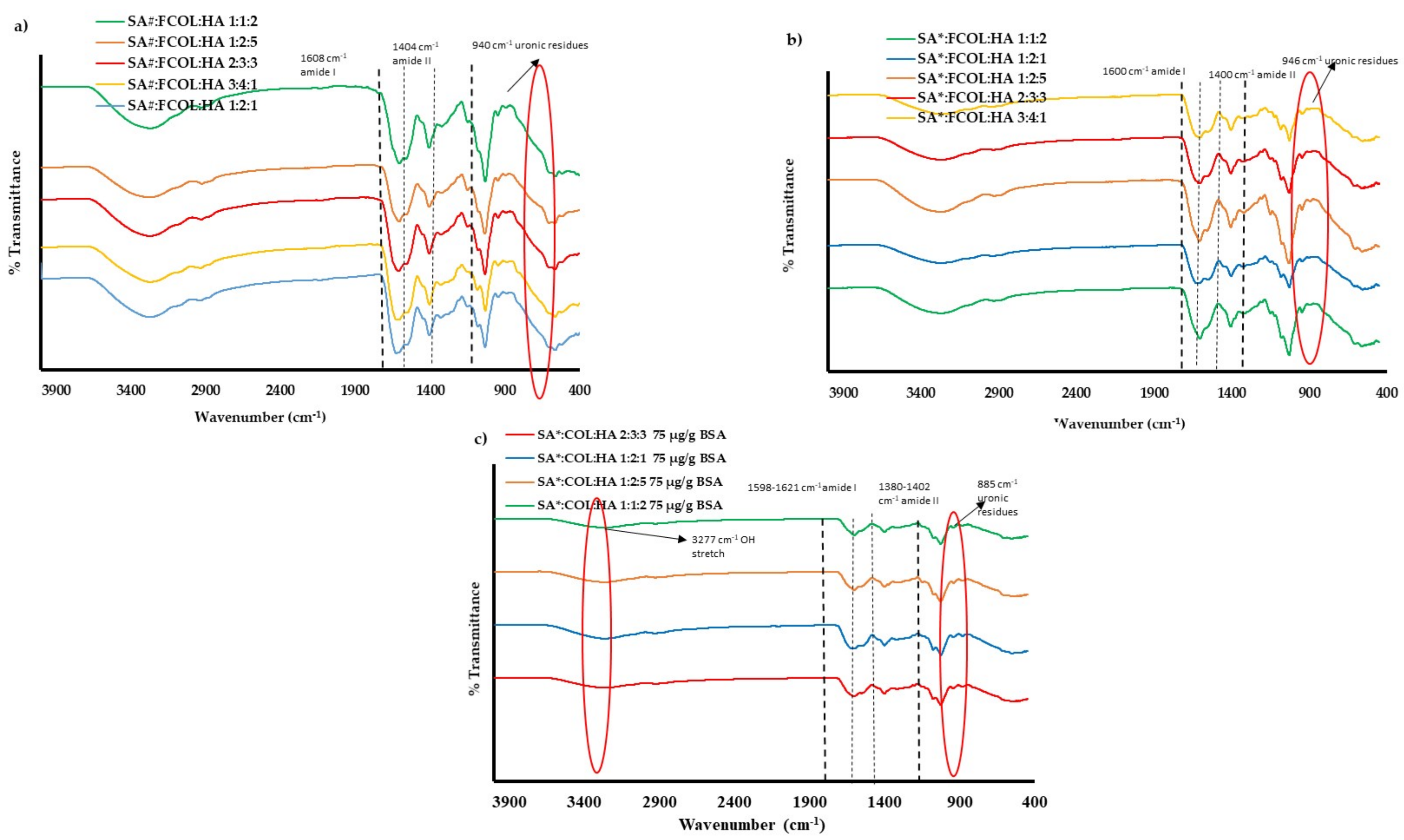

Figure S4. Shows FTIR spectra of (**a**) composite SA#:FCOL:HA and (**b**) SA*: FCOL:HA where the uronic acid residue with higher G content has marginally higher intensity. The amide I and amide II bands appeared to shift toward lower wavenumber, i.e., red shift (from 1635 to 1608 cm^{-1} amide I), (1521 to 1404 cm^{-1} amide II); (**c**) BSA loaded formulations showed further shifts of amide bands, indicating the dispersion of the protein within the polymeric matrix. All the bank shifts observed for the composite formulations are shown in Table S1 of supplementary data.

Table S1. Shows FTIR bands for FCOL, HA, SA and BSA with peak characteristic and assignments. Bands showing shifts within the formulation dressings are highlighted in bold font and their original peaks shown in the right column.

**Dressing component**	**Vibration (cm^{-1}) in composite formulation**	**Peak characteristic and assignment**	**Original peak position (cm^{-1}) in starting material**
**FCOL**	**1608** (no drug)/**1599** (BSA loaded)	Amide I vibration of C=O	1635
	**1404**	Amide II NH bending and CH stretching	1521
	**1235**	Amide III NH and CH stretching	1241
	**2880**	Amide B CH_2 asymmetrical stretching	2929
**SA**	4000-2700	O-H and C-H stretching	
	**1598**	Asymmetric stretching C-H and O-H stretch	1602

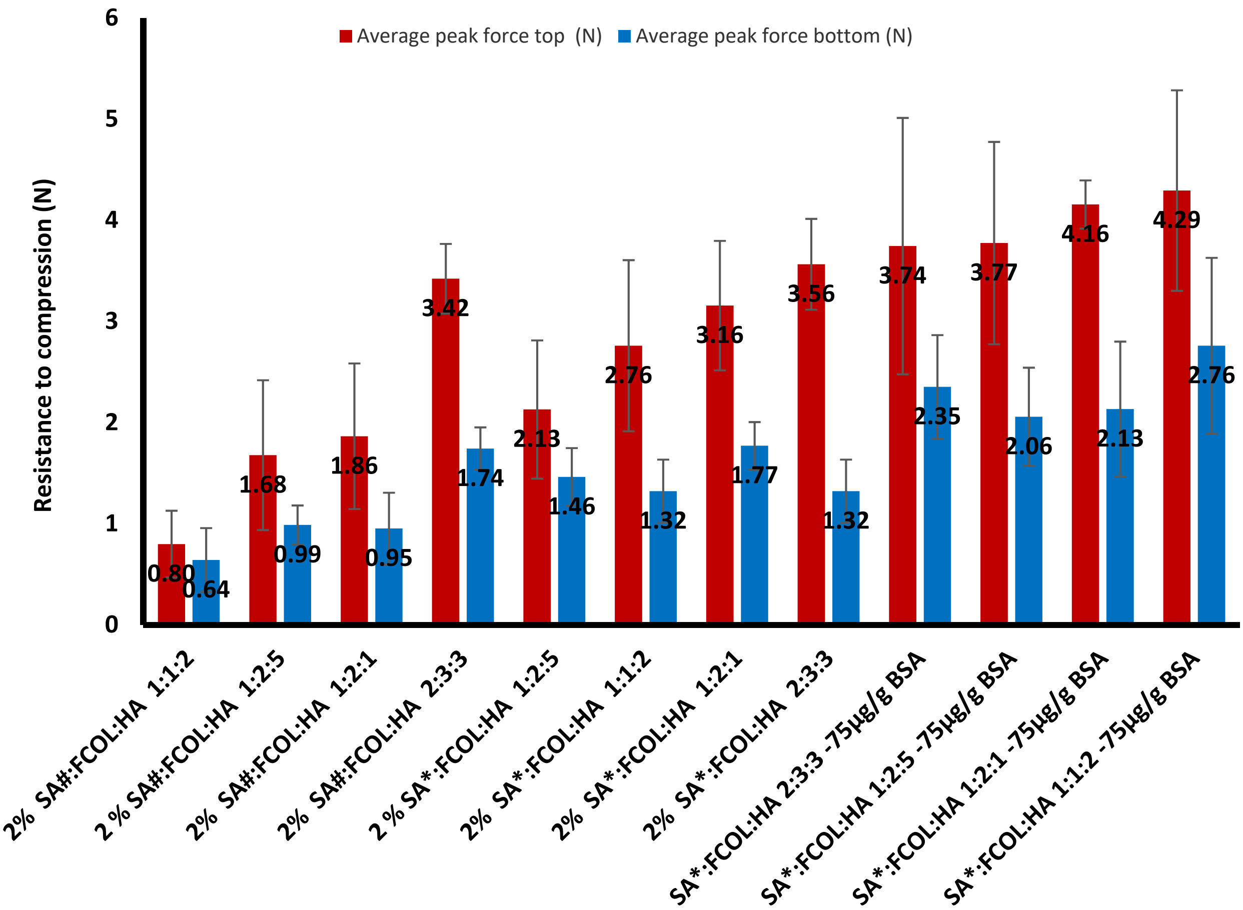

Figure S5. Shows the hardness profile of top and bottom of the formulated scaffolds of 2% SA#:COL:HA, SA*:COL:HA and BSA loaded formulations ($n = 3$). The difference in hardness between the top and bottom of the various scaffolds was significant with $p < 0.0001$ at 95% confidence interval.
